# Physiological characterization of anaerobic cometabolic transformation of sulfamethoxazole by *Nitratidesulfovibrio vulgaris* Hildenborough

**DOI:** 10.1007/s00253-026-13792-3

**Published:** 2026-03-31

**Authors:** Jimmy Köpke, Wei-Ying Ouyang, Caglar Akay, Chang Ding, Aki Sebastian Ruhl, Lorenz Adrian

**Affiliations:** 1 Department Molecular Environmental Biotechnology, Helmholtz Centre for Environmental Research - UFZ, Leipzig, Germany; 2 German Environment Agency, Section II 3.3, Berlin, Germany; 3 Chair of Water Treatment, Technische Universität Berlin, Berlin, Germany; 4 Chair of Geobiotechnology, Technische Universität Berlin, Berlin, Germany

**Keywords:** Activity assay, Bioremediation, Comparative proteomics, Isoxazole ring, Redox differentiation, Retransformation, Sulfate reduction

## Abstract

**Abstract:**

Sulfonamide antibiotics are widely used in medicine and farming and residual amounts end up in wastewater effluents and solids applied as fertilizers in agriculture. Residues could promote antibiotic resistance propagation, especially during activated sludge treatment. We previously reported as a proof of concept that *Nitratidesulfovibrio vulgaris* Hildenborough (NvH) (formerly *Desulfovibrio vulgaris* Hildenborough) can transform the sulfonamide antibiotic sulfamethoxazole (SMX) anaerobically. Here, we studied in detail the influence of SMX on the physiology of NvH, the effect of different electron donors on SMX transformation, and the stability of the transformation products (TPs) under anoxic and oxic conditions. SMX transformation was catalyzed by resting cells and no difference was observed between acclimated and non-acclimated cells. Higher SMX transformation activity was observed with higher initial SMX concentrations and with younger cultures in exponential phase. SMX transformation was supported in the presence of lactate, and slowed down when lactate was depleted. When lactate was replaced with H_2_ plus acetate, sulfate reduction, cell growth, and SMX transformation still took place. The expression pattern of key catabolic proteins was unaltered by the presence of SMX. SMX was transformed by NvH to two major TPs, TP253 and TP255. TP255 was identified as the reduced form of SMX and was abiotically decaying under oxic conditions to TP187, which no longer had an intact sulfonamide structure. Our results suggest that SMX can be transformed under sulfate-reducing conditions by a sequence of anaerobic microbial reduction and oxic abiotic decay, decreasing the persistence and resistance propagation potential of SMX.

**Key points:**

• *Cell components of Nitratidesulfovibrio vulgaris cometabolically transform SMX*

• *Lactate and H*_*2*_* as electron donors promoted SMX transformation*

• *SMX did not lead to significant alteration of the NvH proteome *

**Supplementary Information:**

The online version contains supplementary material available at 10.1007/s00253-026-13792-3.

## Introduction

The high global consumption of antibiotics (Carvalho and Santos 2016; Klein et al. 2018; Van Boeckel et al. 2014) and the persistence of antibiotics in wastewater treatment plants (WWTPs) result in large emission of antibiotics into the environment. Hotspots are WWTP effluents with concentrations in the range of higher ng L^−1^ to middle µg L^−1^ (Ben et al. 2018; Chenxi et al. 2008; Falås et al. 2016; Homem and Santos 2011; Kümmerer 2009a; Onesios et al. 2009; Verlicchi et al. 2012b). These concentrations are considered sub-inhibitory but contributing to the propagation of antibiotic-resistant bacteria and antibiotic resistance genes (Banin et al. 2017; Kümmerer 2009a, 2009b; Rizzo et al. 2013; van Hoek et al. 2011; Zaman et al. 2017). The use of antibiotics as growth promoters in livestock farming amounts to 35–70% of the global prescriptions (Adler et al. 2018; Rosenblatt-Farrell 2009; Zhang et al. 2015). This is regarded to strongly favor resistant bacteria and gene dissemination with the dim prospect of a post-effective antibiotic era (Kraemer et al. 2019; Kümmerer 2009b; O´Neill 2014; Rosenblatt-Farrell 2009; WHO 2017; Zaman et al. 2017).

The sulfonamide sulfamethoxazole (SMX) consists of an amino-substituted benzene ring, which is connected via a sulfonamide bond to a methylated isoxazole ring. SMX is one of the most often used antibiotics in livestock farming and frequently detected in wastewater from the pharmaceutical industry (up to 1.3 mg L^−1^), in hospital wastewater (up to 28 µg L^−1^), and in municipal wastewater (up to 0.9 µg L^−1^) (García Galán et al. 2012; Huber et al. 2005; Verlicchi et al. 2012a; Xu et al. 2007). Moreover, SMX was detected in drinking water (up to 12 ng L^−1^) (Al Aukidy et al. 2012; Kolpin et al. 2002; Morasch et al. 2010; Padhye et al. 2014), rivers and groundwater (up to 11 µg L^−1^) (Cabeza et al. 2012; Dinh et al. 2011; García Galán et al. 2012; Gros et al. 2012; Kibuye et al. 2019), livestock manure (up to 18 mg L^−1^) (An et al. 2015; Ghirardini et al. 2020; Hu et al. 2010), and soil (up to 400 µg kg^−1^ dry matter) (Grossberger et al. 2014; Hu et al. 2010). SMX can even be detected in crops (up to 100 µg kg^−1^ wet weight) (Christou et al. 2017; Franklin et al. 2016; Goldstein et al. 2014; Malchi et al. 2014).

Conventional activated sludge treatment does not efficiently remove SMX from wastewater (Kümmerer 2009a; Verlicchi et al. 2012a, b) as also confirmed by laboratory experiments (Benotti and Brownawell 2009; Cetecioglu et al. 2016; Gartiser et al. 2007; Joss et al. 2006; Li and Zhang 2010; Radke et al. 2009; Yang et al. 2011; Yu et al. 2011). In contrast, anaerobic treatment led more consistently to a SMX removal of 80–100% in laboratory experiments (Alvarino et al. 2014; Aydin et al. 2015; Azimir et al. 2017; Carneiro et al. 2019; Chatila et al. 2016; Fan et al. 2019; Feng et al. 2017; Mohring et al. 2009). Different electron acceptors such as sulfate (Jia et al. 2017; Mohatt et al. 2011; Ouyang et al. 2021), nitrate (Nödler et al. 2012), ferric iron (Mohatt et al. 2011) or carbonate (Cetecioglu et al. 2013; Falås et al. 2016) can be microbially reduced while transforming SMX at varying removal efficiencies (Wang and Wang 2018).

**Fig. 1:**
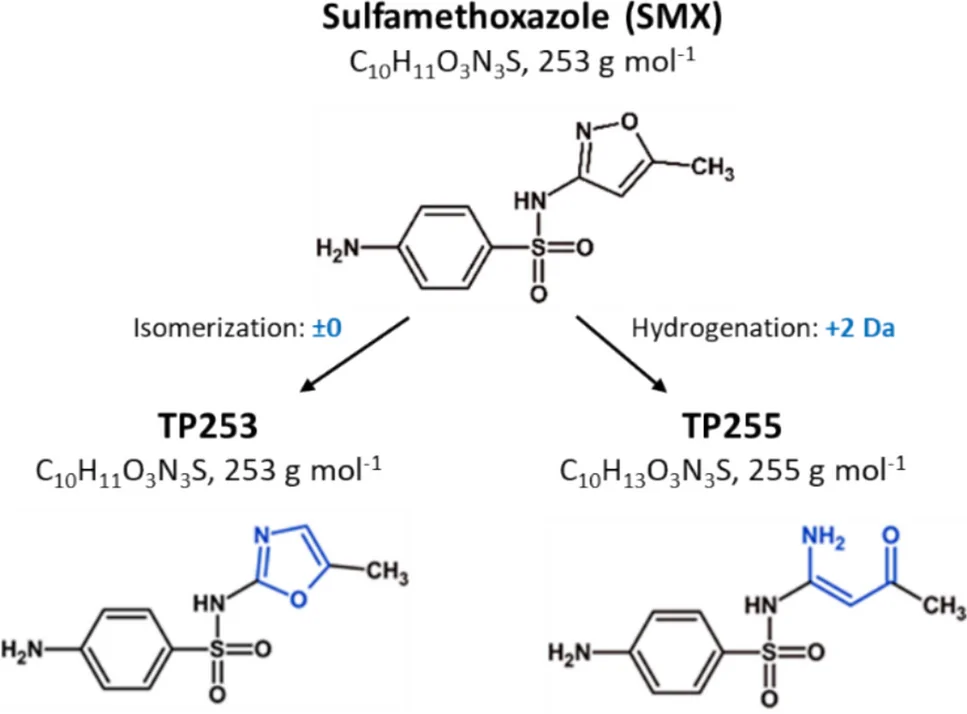
Chemical structure, formula, and molecular mass for SMX, TP253 (SMX-isomer, isoxazole ring-rearranged), and TP255 (dihydrogenated isoxazole ring) (Ouyang et al. 2021)

Under sulfate-reducing conditions, high SMX biotransformation degrees were reported: 60% SMX and 60% of the human metabolite *N*^*4*^-acetyl-SMX were removed in a reactor (Falås et al. 2016); > 95% were transformed within 5 days in soil microcosms (Mohatt et al. 2011); 100% were transformed in laboratory-scale mangrove sediment experiments (Yang et al. 2018); and 100% in fermentation batches (Cetecioglu et al. 2013). Jia et al. (2017) reported SMX biotransformation by sulfate-reducing bacteria in an upflow sludge bed reactor. They proposed several transformation pathways, where most of the transformation products (TPs) had an altered or cleaved isoxazole ring. Similar SMX TPs were found in two of our previous studies under sulfate-reducing conditions in enriched pig-farm impacted soil microcosms (Ouyang et al. 2019) and in enriched digester sludge (Akay et al. 2024). In both studies, we identified TPs altered at the aniline and isoxazole moiety of SMX. Moreover, an initial proof-of-concept experiment with a pure culture of *Nitratidesulfovibrio vulgaris* Hildenborough (NvH) demonstrated SMX transformation and suggested one pathway leading to a TP with a reduced isoxazole moiety (TP255; neutral mass 255) and a second pathway leading to an isomerized oxazole ring (TP253) (Ouyang et al. 2021); Fig. 1).

*N. vulgaris* is well characterized (Heidelberg et al. 2004; Keller and Wall 2011; Pereira et al. 2011, 2008; Pieulle et al. 2005; Vita et al. 2015; Walker et al. 2009) with the complete genome sequenced for strain NvH (Heidelberg et al. 2004). NvH employs cytoplasmic substrate oxidation (lactate, H_2_) and dissimilatory sulfate reduction linked to proton translocation across the membrane generating a proton motive force. Various pathways or mechanisms play a role for energy conservation and are being discussed in the literature including H_2_-based electron transfer (Odom and Peck, 1981), quinone or menaquinone interacting membrane-bound oxidoreductase complexes (Haveman et al. 2004; Pires et al. 2003, 2006). Also discussed are a large pool of type I tetraheme cytochrome c_3_ enzymes (Typ I-c_3_) and fumarate-based electron transfer (Heidelberg et al. 2004; Huang et al. 2021; Tang et al. 2007; Voordouw 2002). Apart from sulfate, NvH is also able to respire with nitrate, Fe(III), U(VI), or Cr(VI) as terminal electron acceptors (Lovley and Phillips 1994; Lovley et al. 1993a, b). The genome of strain NvH encodes proteins of a folic acid biosynthesis pathway including dihydropteroate synthase, which is the target enzyme for the bacteriostatic effect of SMX.

In this study, we used NvH as a model sulfate-reducing bacterium to investigate the anaerobic transformation of SMX, which so far had only been studied with enriched microbial consortia under sulfate-reducing conditions. Specifically, we examined whether SMX transformation is catalyzed by cellular components, whether it supports microbial growth or is cometabolic, and whether SMX and sulfate compete as terminal electron acceptors. We further assessed the influence of electron donor availability (lactate, H_2_) on SMX transformation and evaluated changes in the protein expression profile when NvH was cultivated with SMX and H_2_ plus acetate instead of lactate. Thereby, we aimed to characterize SMX transforming proteins and understand the molecular basis behind this process, which has not been described yet. In addition, we characterized the stability of major SMX transformation products (TP253, TP255) under anoxic-oxic conditions and investigated the formation and kinetics of secondary abiotic TPs. Thereby, we aimed to answer whether an anaerobic treatment step could enhance SMX attenuation during water treatment.

## Material and methods

### Cultivation of *N. vulgaris* Hildenborough (NvH)

*Nitratidesulfovibrio vulgaris* Hildenborough (formerly *Desulfovibrio vulgaris* Hildenborough; DSM 644) was obtained from the German Collection of Microorganisms and Cell Cultures (DSMZ) as a freeze-dried culture. The strain was anaerobically transferred and cultivated in an anoxic medium (Adrian et al. 1998) with 18 mM lactate as electron donor and 21 mM potassium sulfate as electron acceptor at 30 °C in the dark without shaking (text S1). The compositions of the cultivation media are described in Tables S1–S4. The pH value of the 10 mM NaHCO_3_ buffered anoxic medium was at 7.0 and 7.2 before and after incubation and thus should not have affected SMX specification during cultivation (SMX *pK*_*a*1_ = 1.6, *pK*_*a*2_ = 5.7). The culture was transferred to new anoxic medium (3% vol/vol) every 2–3 weeks for maintenance of the active culture. If not stated otherwise, experiments were conducted with 3-day-old cultures as starting inoculum. Growth was assessed by optical density measurements at 600 nm and epifluorescence microscopic counting of cells (text S2). Catabolic activity was assessed by quantification of sulfide.

### Transformation experiment design

The general approach to investigate details about anaerobic SMX transformation by NvH cultures was as follows: NvH cells were provided with an electron donor and an electron acceptor and were exposed to SMX. To better control the flow of electrons from electron donor to acceptor and investigate the role of anaerobic SMX transformation, we incubated cells of different cultivation stages (stationary phase or exponential), with artificial and physiological electron donors (methyl viologen, lactate, H_2_), physiological or potential electron acceptors (sulfate, SMX), and at varying concentrations of the components. All transformation experiments were conducted in triplicate, statically at 30 °C in the dark with specific cultivation conditions and monitored parameters as summarized in Table 1.

**Table 1:** Overview of conducted batch experiments including experimental setup, initial conditions, and monitored parameters

Experiment #	Initial SMX/TPs	Electron donor/C-source	Electron acceptor	Incubation time	Monitored compounds	Incubation conditions
1	100 µM	1 mM methyl viologen (reduced)	100 µM SMX	4 days	SMX, TP253, TP255	Exponential-phase cells, anoxic
2	50–650 µM	18 mM lactate	20 mM sulfate	28 days	SMX, TP253, TP255, cell density, sulfate, sulfide	Stationary-phase cells, anoxic
3	100 µM	18 mM lactate	20 mM sulfate	23 days	SMX, TP253, TP255, cell density, sulfide	Exponential-phase cells, anoxic
4	100 µM	8–40 mM lactate	8 mM sulfate	229 days	SMX, TP253, TP255, cell density, lactate, acetate, sulfide	Exponential-phase cells, anoxic
5	100 µM	16 mM lactate; 32 mM eq H_2_, 2 mM acetate	8 mM sulfate	33 days	SMX, TP253, TP255, cell density, lactate, H_2_, acetate, sulfide, protein expression	Exponential-phase cells, anoxic
6	50 µM SMX/14 µM TP253/54 µM TP255	-	-	35 days	SMX, TP253, TP255, Unknown TP formation	No cells, oxic

Averages of triplicates and standard deviations are reported for all monitored parameters. Significant changes of abiotic controls were tested by repeated-measures two-way analysis of variance (two-factor ANOVA without replication). The effect of different SMX or lactate concentrations on sulfide production, cell growth, or SMX transformation rates was tested applying mixed-effects models with and without replicate-specific slopes using likelihood-ratio tests. For all statistical analysis, significance criteria were set to 0.05 (*p*-value). Analyses were performed in R (version 4.4.2) using the lme4 and lmerTest packages.

### Quantification of SMX and its transformation products

Concentrations of SMX and TPs were determined by ultra-performance liquid chromatography (UPLC) attached to a photo diode array detector (DAD) (Thermo Fischer UltiMate 3000 RS Diode Array Detector) with a C18 column (LiChrospher 100, RP-18, endcapped, 5 µM; Merck) (Ouyang et al. 2021). SMX was analyzed directly from sterile-filtered culture medium (0.2-µm cellulose acetate filter) of 200-µL samples in inserts of 1.5-mL glass vials. For each measurement, 10-µL samples were injected at room temperature and absorption was quantified at 269.5–270.5 nm. The chromatography was performed using a flow rate of 0.6 mL min^−1^ and a gradient method based on a mobile phase of water (0.1% vol/vol formic acid, solvent A) and methanol (solvent B). The gradient method was as follows: 0–2.0 min (5% solvent B), 1.25–8.0 min (5–98% B), 8.0–10.0 min (98% B); 10.0–17.0 min (98–5% B). SMX quantification was done with a linear regression model (R^2^ = 0.996) based on external SMX calibration standards in acetone. Since the UV-active aminobenzene moiety of identified TPs was still intact, similar absorption coefficients for TPs as for SMX were assumed. Hence, the same linear regression model was applied for estimation of TP quantities. The limit of detection and limit of quantification were 100 nM and 0.5 µM SMX (or TPs), respectively.

### Identification of transformation products of SMX

Characterization of secondary TPs generated from oxic incubation of the TPs generated by anaerobic microbial transformation of SMX was carried out by an Agilent 1260 Infinity II Series liquid chromatograph (LC) system coupled to an AB SCIEX QTRAP® 6500+ tandem mass spectrometer (MS/MS) equipped with a Turbo V ion source. The system was operated with an electrospray ionization (ESI) probe in positive polarity. For compound separation we used a Zorbax Eclipse Plus Rapid Resolution HT-C18 column (100 mm × 3.0 mm, 1.8 µm) attached to a Phenomenex security guard cartridge system (C18; ODS, Octadecyl). The injection volume was 50 µL, the column temperature was set at 30 °C, and the flow rate was at 0.4 mL min^−1^. The binary mobile phase consisted of 0.2% (v/v) formic acid in LC-MS grade water (solvent A) and LC-MS grade methanol (solvent B). The LC gradient program was as follows: 0–2 min (10% of B), 2–3 min (10–60% of B), 3–8 min (60–90% of B), 8–11 min (90% of B), and 11.1–16 min (10% of B). The ion source–dependent MS parameters for identification and structure verification of SMX and known TPs are described in text S3 of the supplementary information.

To investigate SMX TP stability, samples from the end of incubation experiments of NvH and initial 100 µM SMX were filtered through 0.2-µm pore size filters to remove particles. The filtrate was aerobically shaken for 30 min at 200 rpm. A volume of 300 µL of the oxidized, cell-free filtrate was injected to closed 1.5-mL amber glass vials, at room temperature, in triplicate and in the dark. Each vial was opened once a day for 30 s to allow gas exchange. For each sampling point, triplicate samples were sacrificed.

The screening of unknown TPs was performed using a precursor ion survey scan that generated a typical sulfonamide fragment ion of *m/z* 156. The precursor ion scan was combined with the information-dependent acquisition (IDA) criteria set at an intensity threshold of 50,000 counts per second, followed by an enhanced product ion (EPI) scan as the dependent scan. When a precursor ion was detected above the specified intensity threshold, an EPI scan was triggered to acquire a full-scan MS/MS spectrum. The obtained fragments within MS^2^ were interpreted for structure elucidation. All acquired data was processed using the Analyst 1.7.1. software.

### Determination of sulfate, sulfide, lactate, acetate, and H_2_

Sulfate concentrations were assessed using a Dionex DX-120 ion chromatograph equipped with a Dionex RIFC IonPac AS4A-SC (4 × 250 mm) analytical column and a Dionex RFIC IonPac AG4A-SC (4 × 50 mm) guard column. A mobile phase of 4.5 mM Na_2_CO_3_ and 1.4 mM NaHCO_3_ at a flow rate of 1 mL min^−1^, and an isocratic gradient for 10 min resulted in a retention time of 4.60 min for sulfate. Sulfide was quantified using absorbance at 480 nm (Thermo spectral photometer UV-Vis, Evolution 160) after reaction of sulfide with copper to colloidal CuS in 5 mM CuSO_4_ and 50 mM HCl in a sample to buffer ratio of 1:5 (v/v) as described by Cord-Ruwisch (1985). Lactate and acetate concentrations were determined by UPLC-DAD (Thermo Fischer UltiMate 3000 RS diode array detector) with a Rezex ROA-organic acid column (H^+^ (8%); 150 × 7.8 mm; Phenomenex) and Rezex ROA-Organic Acid LC guard column (H^+^ (8%), 50 × 7.8 mm, Phenomenex) using an isocratic elution of 2.5 mM H_2_SO_4_, a flow rate of 0.5 mL min^−1^, and 10 µL injection volume. Absorption was monitored at 210 nm. Calibrations of all compounds were done with external standards in anoxic water with correlation coefficients R^2^ above 0.98. Detection limits were 0.5 and 0.2 mM for lactate and acetate, respectively.

H_2_ consumption was followed using an analog pressure sensor (MPX5100DP, NXP Semiconductors, Eindhoven, Netherlands) monitoring the headspace pressure within the incubation bottles after over-pressurizing them with pure H_2_ gas. The analog signal was converted to a digital signal using an analog-to-digital converter (T7Pro, LabJack, USA). H_2_ was quantified (in mL) by comparing measured headspace pressure within the incubation bottles (in mV) to known volumes of H_2_ spiked into the same bottles with the same headspace and liquid volume. The air pressure during each sampling event and loss of headspace pressure during sampling was a priori determined and accounted for using a correction factor for quantified H_2_ volumes (in mL). Nominal concentrations of H_2_ (mM) in the aqueous phase were calculated by dividing the H_2_ amount (in mmol) by the liquid volume.

### Whole-cell in vitro activity assay

The biochemical basis of the whole-cell in vitro activity assay was the following: electrons from the reducing agent Ti(III)citrate are stoichiometrically channeled to methyl viologen forming radicals. These radicals can be used as artificial electron donor by NvH cells to reductively transform SMX. Hence, the whole-cell in-vitro activity assay was conducted by pre-mixing methyl viologen and Ti(III) citrate to 1 mM each, whereby Ti(III)citrate abiotically reduces methyl viologen first. Lastly, SMX was added at 100 µM into 2-mL glass vials to avoid abiotic SMX transformation by Ti(III)citrate (Table S4). Concentrated whole cells of NvH (1.5 × 10^9^ cells mL^−1^) were harvested from an exponential growth phase (3 days old) or stationary phase (11 days old) culture via three rounds of centrifugation at 6000 × *g* and washing with 200 mM potassium phosphate buffer. Whole cells were added to the reaction mix under anoxic conditions, and the closed vials were incubated statically at 30 °C in the dark.

### Comparative shotgun proteomics

Crude extracts of strain NvH were analyzed using shotgun proteomics. Cells from NvH were harvested at late stationary phase on day 31 of incubation and tryptically digested, derivatized, purified, and analyzed on a nanoHPLC system (Dionex Ultimate 3000RSLC, Thermo Fisher Scientific, USA) coupled to an Orbitrap Fusion Tribrid mass spectrometer (Thermo Fisher Scientific, USA). The methodology was previously described by Ding and Adrian (2020) but was modified as described in detail in text S4. Peptides in 6-µL injected samples were separated at 35 °C on a 15-cm analytical column (Acclaim PepMap RSLC, 3 µm × 250 mm, C18 particles, Thermo Scientific) using a 120-min gradient from 4 to 55% solvent A (0.1% formic acid) to solvent B (80% acetonitrile, 0.08% formic acid) and a 300-nL min^−1^ flow rate. Peptides were ionized using a TriVersa NanoMate, Advion electrospray ion source in positive mode. Precursor ions were measured in the Orbitrap analyzer at a resolution of 120,000; fragmentation was done at 30% collision-induced dissociation and fragments were measured in the ion trap at a resolution of 30,000. Only precursors with a charge state between 2 and 4 were selected for fragmentation. Protein identification and statistical analysis was carried out using Proteome Discoverer 2.4 (Thermo Fisher Scientific, USA), and fragment mass spectra (MS^2^) were compared using the SequestHT search engine against genome encoded proteins of NvH (NCBI accession number GCA_000195755.1). The proteomics data has been deposited to the ProteomeXchange Consortium via the PRIDE partner repository with the dataset identifier PXD068774 and /10.6019/PXD068774. Comparative proteomics were performed on two differently grown cultures. Protein abundance ratios were calculated based on pair-wise comparison of each replicate among triplicates of each culture. Maximum allowed protein fold changes were set to 100 and missing peptide signals in some of the triplicates were imputated via replicate-based resampling (random values sampled from distributions around the medians of detected values of replicates). Statistical significance was calculated using a background-based *t*-test with Benjamini-Hochberg correction (Benjamini and Hochberg 1995) and the adjusted significance thresholds were set to 0.05.

## Results

### SMX transformation was catalyzed by cell components of NvH

**Fig. 2:**
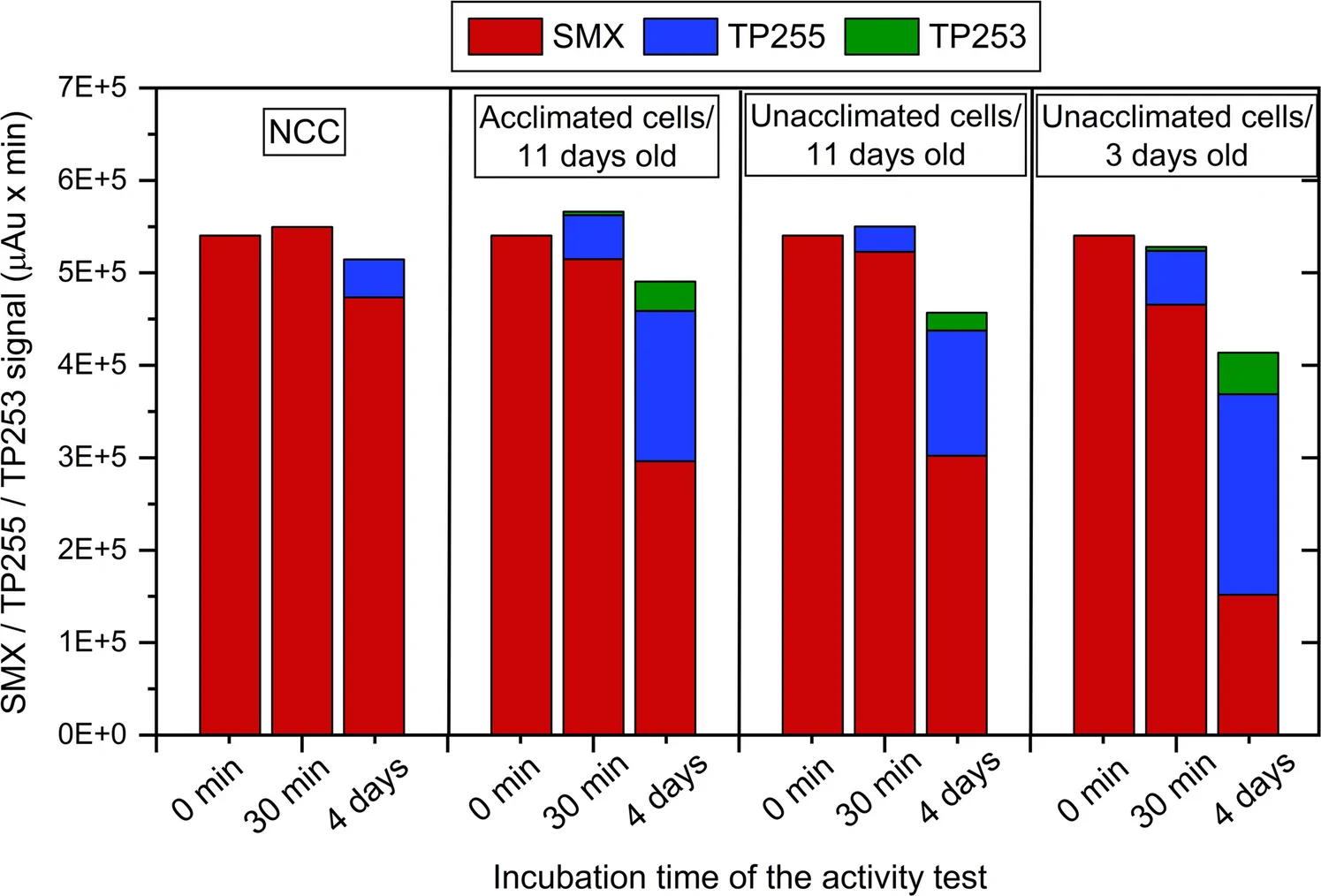
Transformation of SMX and production of TP255 and TP253 by whole cells of NvH within in vitro activity assays using reduced methyl viologen as electron donor. The initial SMX concentration was 100 µM and the cell density in the tests was 1.5 × 10^9^ cells mL^−1^. Shown are no-cell controls (NCC) without NvH cells and vials that were inoculated with cultures from different growth phases: 11 days growth with 100 µM SMX (acclimated), 11 days growth without SMX (unacclimated), 3 days growth without SMX (unacclimated)

Whole-cell activity tests with reduced methyl viologen as an abiotic electron donor and SMX as electron acceptor were performed using cells in exponential phase (3-day-old cells) or stationary phase (11-day-old cells) (Fig. 2). All culture sets contained lactate and sulfate. Reduced methyl viologen alone (no-cell control, NCC) did not majorly transform SMX. Incubation of whole cells in the presence of reduced methyl viologen and SMX resulted in two TPs (TP255, TP253) with the *m/z* values of 255 and 253, respectively. The transformation of SMX to TP255 (5.8 × 10^4^ µAu × min) and TP253 (4.3 × 10^3^ µAu × min) was already evident at the 30-min sampling point, showing a rapid onset of biotransformation. By day 4, the levels of TP255 and TP253 had increased to 2.2 × 10^5^ and 4.5 × 10^4^ µAu × min, respectively. The transformation of SMX to TP255 and TP253 was dependent on the presence of NvH cells and the growth phase, with cells from the exponential phase (3-day-old cells) showing faster SMX transformation (Fig. 2). No considerable difference in the SMX transformation rate between cells grown in the presence of 100 µM SMX and cells grown without SMX was observed. These results indicated that the activity was not specifically induced by SMX (Fig. 2).

### Effect of SMX concentration and culture stage on SMX transformation

**Fig. 3:**
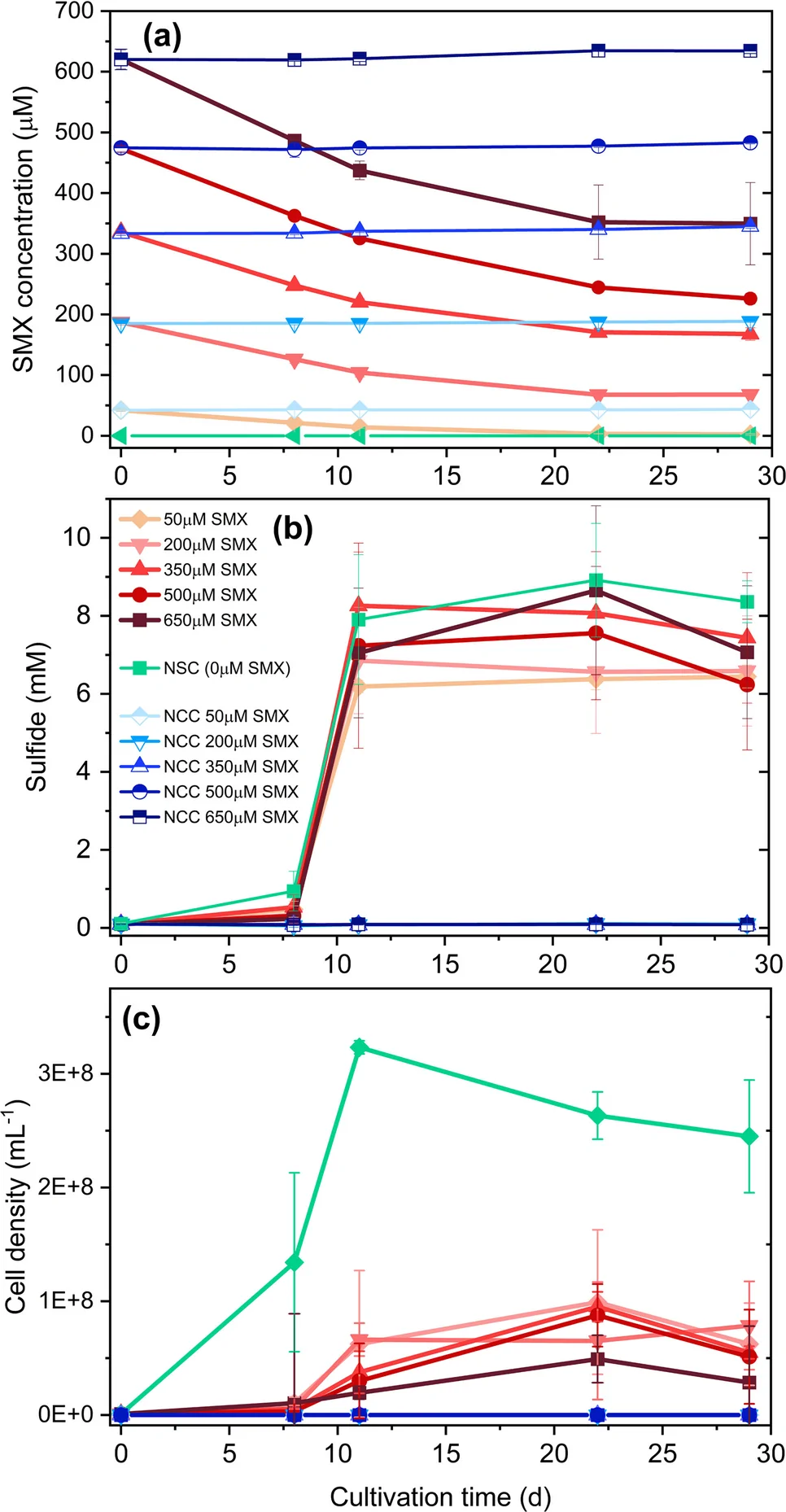
Effect of different SMX concentrations on the physiology of NvH. Cultures of NvH were inoculated with cells from the stationary phase containing 18 mM lactate, 20 mM sulfate, and different initial concentrations of SMX. Shown are **a** SMX concentrations over time, **b** sulfide concentration, and **c** cell density. Controls without cells but with SMX (NCC, blueish colors) and with cells but without SMX (NSC, green) were included. The initial cell density was 1.1 × 10^6^ cells mL^−1^, except for the NCCs were no cells were observed (lines are overlapping)

**Fig. 4:**
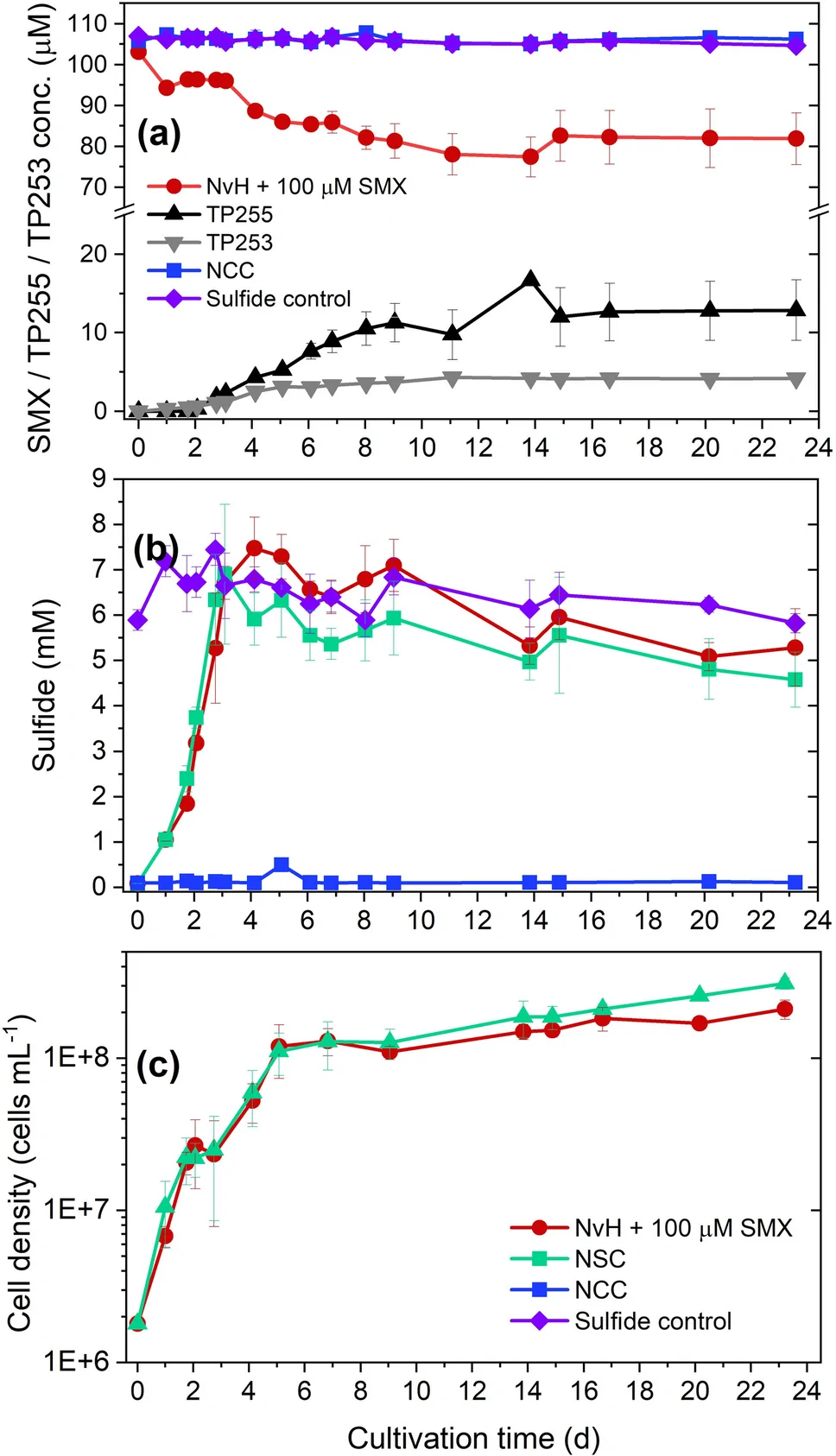
Formation of TPs during the cultivation of NvH. SMX conversion, sulfide production as an indicator for sulfate reduction, and growth in cultures of NvH incubated are shown. Cultures contained 18 mM lactate, 20 mM sulfate, and cells from the exponential phase (3 days culture). Shown are **a** SMX and TP concentrations, **b** sulfide concentrations, and **c** cell density. Cultures were inoculated to an initial cell density of 1.8 × 10^6^ cells mL^−1^

Initial experiments with NvH cells from the stationary phase (14-day-old cultures) showed that cultures with 100 µM SMX, 18 mM lactate, and 20 mM sulfate were able to reduce sulfate stoichiometrically to sulfide. SMX decreased growth of the cells but did not completely inhibit it (Fig. S1). We therefore investigated in more detail the effect of different concentrations of SMX from 50 to 650 µM with cultures inoculated from a stationary phase culture amended with 18 mM lactate and 20 mM sulfate. We decided for a concentration range higher than most reported environmental concentrations to better identify SMX TPs and to induce larger differences in protein expression profiles. The protein expression profiles were investigated to identify SMX transforming proteins within NvH cells. Both sulfate and SMX were transformed at all tested SMX concentrations. Depending on the SMX concentration, between 50 and 82% of the initial SMX was transformed within 28 days of incubation (Fig. 3a). In control bottles without inoculum (NCC), SMX was not transformed. In all bottles, the absolute transformation rate decreased over time and almost no further SMX was transformed after 22 days of incubation (Fig. 3a; Fig. S2). For the initial transformation of 350 to 650 µM SMX, first-order reaction kinetics applied (Table S5). However, SMX transformation depended on sulfate reduction and stalled after day 12. Also, for the 350 to 650 µM SMX cultures, normalized SMX transformation revealed similar relative SMX transformation rates independent from initial SMX concentrations (Fig. S3). Neither sulfate reduction to sulfide nor transformation of SMX was completely inhibited by SMX concentrations up to 650 µM. However, SMX diminished growth of NvH as evidenced by stronger cell growth in control bottles without SMX (no SMX control, NSC; Fig. 3c). Overall, SMX posed a strong inhibitory effect on growth but no significant (*p* = 1.00) effect on sulfate reduction to sulfide (Fig. 3b). SMX cultures did not differ significantly in cell densities (*p* = 0.96). For cultures with 100 µM SMX, 18 mM lactate, 20 mM sulfate, and inoculum from a culture in its exponential phase (3-day-old cultures), growth and sulfate reduction were faster and sulfide levels reached their maximum after 4 days (Fig. 4). Slowly decreasing sulfide levels during the stationary phase can be explained by a loss of sulfide during frequent sampling. SMX transformation resulted in the production of almost equal amounts of TP255 and TP253 within the first 4 days. Afterwards, the conversion of SMX to TP255 continued whereas the formation of TP253 almost stalled. Growth was correlated with the production of sulfide but slow growth continued even after sulfide concentrations had reached their maximum. No SMX transformation occurred within the sulfide control spiked with 100 µM SMX and 8 mM sodium sulfide testing for potential abiotic SMX transformation catalyzed by sulfide. Given that the growth stage influences SMX transformation, all subsequent cultures described in this study were inoculated with cells harvested from the exponential phase, unless stated otherwisel.

### Effect of lactate availability on SMX transformation

Subsequently, the stoichiometry between lactate consumption, sulfate reduction, and SMX transformation was determined during substrate-limiting conditions. All cultures were spiked with 100 µM SMX, which should not have an inhibitory effect on the inoculated culture in the exponential growth state. To avoid toxic effects of the accumulating sulfide, we chose 8 mM of sulfate for long-term incubation. Based on the stoichiometric relation of 2:1 for lactate:sulfate, we amended between 8 and 40 mM of lactate to the cultures allowing either lactate or sulfate to be in excess.

**Fig. 5:**
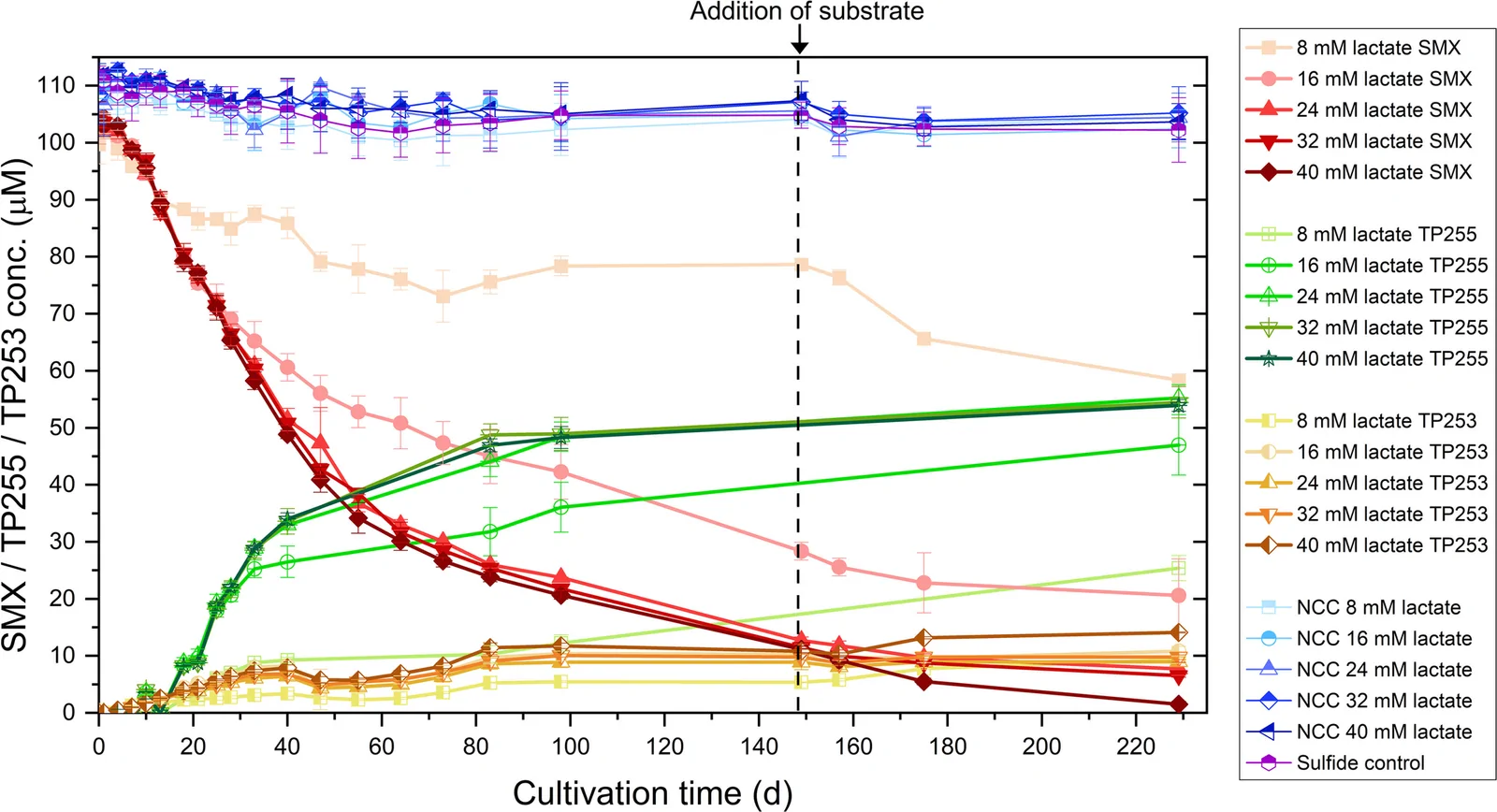
Transformation of SMX (red colors) to TP255 (green colors) and TP253 (yellow colors) by NvH over time at lactate concentrations from 8 to 40 mM. All cultures contained 8 mM sulfate and 100 µM SMX. Controls included NCC (blue colors) and sulfide control (purple). On day 149 (vertical black dashed line), additional 16 mM lactate was added to the 8 mM lactate cultures, 0.3 bar H_2_ (32 mM nominal concentration) to the 16 mM lactate cultures, and 8 mM K_2_SO_4_ to the 40 mM lactate cultures. The samples of days 40–78 and day 98–179 were stored aerobically at 4 °C and −20 °C, respectively, for up to 4 weeks before analysis, while all other samples were measured immediately after sampling

Both TPs, TP255 and TP253, were observed within 6 days of incubation (Fig. 5). SMX transformation slowed down first in the cultures with 8 mM lactate (on day 21) and then within the 16 mM lactate cultures (on day 32). At the same time, lactate became limiting in both cultures (0 mM on day 4; data not shown). The SMX transformation rate per cell was not dependent on the initial concentration of amended lactate (*p* = 1), but seemed to be the highest between days 7 to 13 before decreasing again (Fig. S4). The high initial SMX transformation rates per cell (day 0 to 1) were considered to be insignificant due to highest standard deviations of lowest cell densities (*p* = 1) and thus not further considered (Fig. S4). On day 149, SMX concentration was decreased by 21% (8 mM lactate), 72% (16 mM lactate), 87% (24 mM lactate), 88% (32 mM lactate), and 89% (40 mM lactate). Almost equal amounts of TP255 and TP253 were formed within the first days of incubation. However, after about 15 days of incubation, the formation of TP255 dominated and the formation of TP253 almost stalled. This resulted in a ratio of about 4.3 of TP255/TP253 at the end of the incubation. Due to loss in signal of TP255 for stored samples, data points between days 40 and 83 as well as days 98 and 149 were not included (Fig. 5). However, TP255 formation trends during the whole course of experiment were confirmed.

In all cultures, cell growth, lactate, acetate, and sulfide were monitored and showed no difference between SMX and NSC cultures (Fig. S5). On day 149, no lactate was detected in the cultures with 8 or 16 mM initial lactate but sulfate was still present. To determine the effect of additional electron donors, we amended an additional 16 mM lactate to the 8 mM lactate cultures and added a pressure of 0.3 bar H_2_ (32 mM H_2_ nominal concentration) to the cultures with initially 16 mM lactate. In cultures with initial 40 mM lactate where lactate was still present but sulfate was used up (Fig. S5C), an additional 8 mM sulfate was added on day 149. The analyses in the following 75 days showed that addition of lactate to the 8 mM cultures stimulated SMX transformation and sulfate reduction (Fig. S5G). Contrarily, H_2_ addition to the 16 mM cultures did not have a noticeable effect. The addition of sulfate to the 40 mM lactate cultures resulted in further promotion of SMX transformation and continued sulfate reduction in comparison to the 16 to 32 mM lactate cultures. In summary, sulfate reduction with lactate as electron donor promoted SMX transformation. For an estimated mass balance, the same UV absorption coefficients for SMX and the two TPs were assumed and thus the same linear regression model was applied for quantitation. For the 40 mM lactate cultures, 99% of the initial 100 µM SMX were transformed; however, only an estimated 54 µM TP255 and 14 µM TP253 were detected, representing 65% of the transformed SMX. None of the controls showed considerable SMX transformation or TP formation within 229 days of incubation. The statistically significant yet overall minor decrease in SMX concentrations (*p* < 0.05) observed in the NCCs and sulfide controls was considered negligible as it corresponded to less than a 5% reduction of the initial SMX concentration. Contrarily, the biological cultures exhibited SMX transformation rates ranging from 40 to 99%. Also, no TPs were detected in the controls (data not shown).

### Effect of H_2_ as electron donor on SMX transformation

**Fig. 6:**
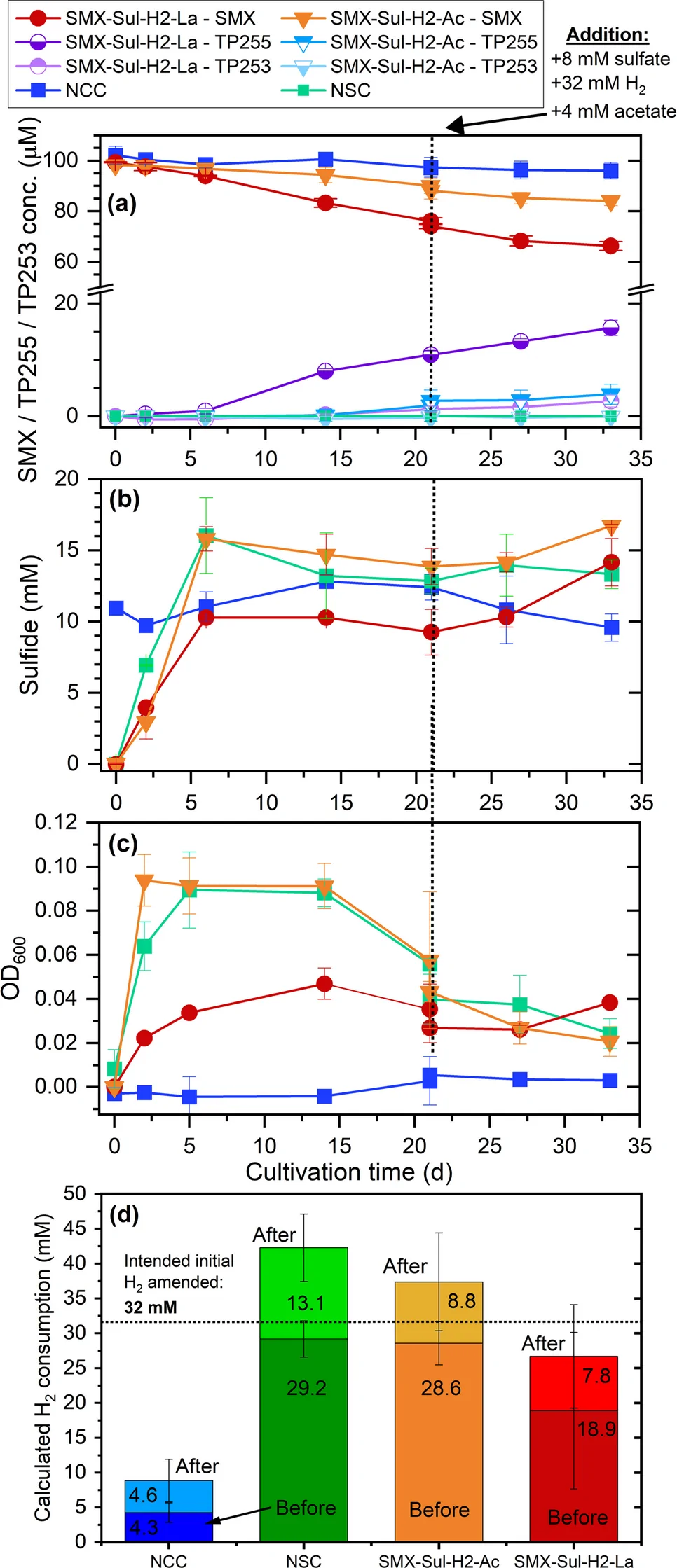
NvH cultures inoculated from an exponential phase culture and grown on acetate, H_2_, and sulfate. Shown are **a** SMX conversion to TP255 and TP253, **b** sulfide production as an indicator for sulfate-reducing activity, **c** cell growth as OD_600_, and **d** H_2_ consumption based on headspace pressure. Included are NCC, NSC, and two SMX cultures further incubated with 8 mM sulfate, and either 32 mM H_2_ and 4 mM acetate (SMX-Sul-H_2_-Ac) or 16 mM lactate (SMX-Sul-H_2_-La). The starting cell density was 1.2 × 10^6^ cells mL^−1^. “Before” and “After” refer to the additional amendment of 8 mM sulfate, 32 mM H_2_, and 4 mM acetate on day 21

NvH was reported to be able to use H_2_ as an electron donor and acetate as a carbon source to metabolically reduce sulfate in a stoichiometric ratio of 4:1 for H_2_ and sulfate (Keller and Wall 2011; Pereira et al. 2011; Vita et al. 2015). We cultivated NvH with 32 mM H_2_ (nominal concentration), 2 mM acetate, and 8 mM sulfate for several transfers before the experiment. The capability of NvH to transform SMX in the presence of H_2_ was tested by inoculating the H_2_ pre-cultured cells to medium with 100 µM SMX, 8 mM sulfate, 32 mM H_2_, and either 16 mM lactate (SMX-Sul-H_2_-La) or 4 mM acetate (SMX-Sul-H_2_-Ac). NCC (no NvH cells) and NSC (no SMX) controls were included in the experiment (Fig. 6).

SMX was transformed to TP255 with a reduced isoxazole ring and TP253 as SMX-isomer in both SMX-Sul-H_2_-La and SMX-Sul-H_2_-Ac, but not in the no-cell control (NCC). However, SMX-Sul-H_2_-La cultures showed faster and overall more SMX transformation (34% of initial 100 µM SMX) than the SMX-Sul-H_2_-Ac without additional lactate (14%, Fig. 6a). We estimated a mass balance using the same regression model for SMX and both TPs. For the SMX-Sul-H_2_-La cultures and 34 mM SMX transformed, we detected 16 mM TP255 and 3 mM TP253 thus in total 18 mM (55% of 34 mM SMX loss). For the SMX-Sul-H_2_-Ac and 14 mM transformed SMX, we detected 3.9 mM TP255 and no TP253. Sulfide production, cell growth, and H_2_ consumption were less in the SMX-Sul-H_2_-La than in SMX-Sul-H_2_-Ac cultures and the no SMX control (NSC) (Fig. 6d). This can be explained by the inoculum having been cultivated for several generations on H_2_ and acetate. The calculated H_2_ consumption had been equally high in the NSC and SMX-Sul-H_2_-Ac cultures (about 29 mM H_2_), but lower in the SMX-Sul-H_2_-La cultures (about 19 mM H_2_) where 16 mM lactate as additional electron donor was spiked. The monitored headspace pressure to calculate H_2_ consumption by NvH is shown in Fig. S6. The addition of 32 mM H_2_, 4 mM acetate, and 8 mM sulfate on day 21 further promoted sulfide production for both electron donor cultures but not cell growth or further SMX transformation (Fig. 6).

Using shotgun proteomics, we detected key catabolic proteins involved in lactate or H_2_ oxidation, electron conduction, and dissimilatory sulfate reduction (Table S5). For the lactate oxidation pathway, we detected *L*-lactate dehydrogenase (LdH), *L*-lactate permease (LtP), acetate kinase (AckA), formate dehydrogenase (FdhB/D/E), and pyruvate ferredoxin oxidoreductase (PorA/B). For H_2_ oxidation, we detected the membrane-bound cytoplasmatically oriented energy-converting hydrogenase (EchA/C/D/E/F), high molecular mass cytochromes (HmcA/B/C/F), hydrogenase maturation proteins (HypA1/A2/D/E), periplasmic [Fe] hydrogenase (HydA/B), periplasmic [NiFe] hydrogenase (HynA-1/2), and periplasmic [NiFeSe] hydrogenase (HysA/B). Identified proteins involved in dissimilatory sulfate reduction included adenylsulfate reductase (AprA/B), sulfate adenyltransferase (Sat), sulfate permease (SulP), and dissimilatory sulfite reductase (DsrA/B/C/D/J/K/O/P). Proteins that are involved in electron channeling as for instance coupling lactate or H_2_ oxidation to sulfate reduction included cytochrome c, the electron transport complex (RnfB/C/E/G), menaquinone reductase (QrcB/C/D), some iron-sulfur cluster binding proteins, and ferredoxins (Table S5). Significantly higher expression of lactate permease proteins (LtP, dvu3026) in cultures grown with lactate instead of H_2_ as electron donor was as expected.

### Comparative shotgun proteomics of NvH grown with and without SMX

**Fig. 7:**
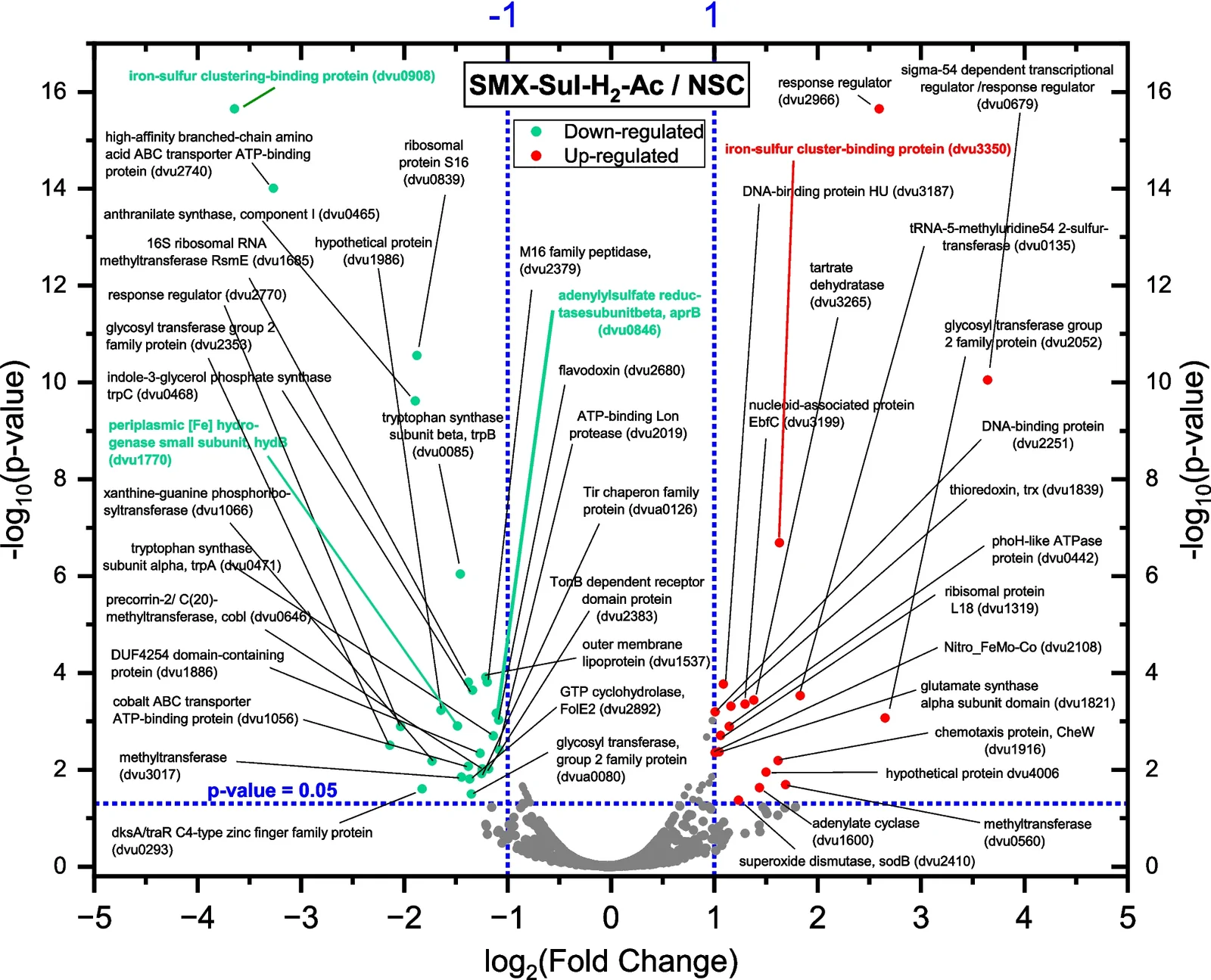
Volcano plot showing the difference in protein abundance between cultures of NvH grown on acetate and H_2_ with or without 100 µM SMX (SMX-Sul-H_2_-Ac vs. NSC)*.* The x-axis shows the log_2_ (fold change) in the protein abundance ratio SMX-Sul-H_2_-Ac/NSC. Highlighted are down- (green) or up-regulated proteins (red) with a log_2_ (fold change) below or above −1 or 1 that met the set significance criteria shown as −log_10_ (*p*-value ≤ 0.05). Further discussed candidates are highlighted in green or red

We performed comparative proteomics to identify specifically induced proteins when SMX was added to the culture. For this, we set up cultures of NvH grown on acetate and H_2_ with and without 100 µM SMX and compared protein abundances (SMX-Sul-H_2_-Ac vs. NSC; Fig. 7). Here, we define up-regulation as higher protein abundance within the cultures with SMX and down-regulation vice versa. Most proteins were not significantly down- or up-regulated (*p* ≥ 0.05; fold change ≤ 2). Most proteins with a fold change ≥ 1 are not predominantly involved in key catabolic processes, but different cellular mechanisms. Exceptions were the adenylsulfate reductase subunit beta (AprB, encoded by dvu0846), the small subunit of periplasmic [Fe] hydrogenase (HydB, encoded by dvu1770), and an iron-sulfur cluster binding protein showing the highest fold change and significance (Fig. 7). Both cultures with and without SMX produced similar amounts of sulfide, indicating the down-regulation of AprB did not significantly (*p* > 0.05) affect the catabolic reduction of sulfate to sulfide (Fig. 6b). The same applies to the periplasmic [Fe] hydrogenase protein HydB (encoded by dvu1770), which was reported to be involved in an electron transport chain during H_2_ uptake for catabolic sulfate reduction with cytochromes c3 likely being the physiological electron carrier (Keller and Wall 2011; Pereira et al. 2011, 2008; Pieulle et al. 2005; Vita et al. 2015; Walker et al. 2009). HydB was slightly down-regulated with SMX but both cultures had similar H_2_ consumption. In contrast, the iron-sulfur cluster binding protein (encoded by dvu0908) was strongly down-regulated but its presumed electron transfer role in the metabolism is unknown.

Similarly, most of the proteins up-regulated in the SMX culture did not have a fold change above 2, although the difference was statistically significant (*p* ≤ 0.05). One iron-sulfur cluster binding protein (encoded by dvu3350) was significantly up-regulated, however is only annotated in another *Nitratidesulfovibrio vulgaris* strain (UniProt). Thereby, it is annotated as 2-oxoglutarate ferredoxin oxidoreductase protein. The clear presumed electron transfer role of that protein in the metabolism of NvH is however still not clear. Some of the other significantly up- or down-regulated proteins such as superoxide dismutase (encoded by dvu2410) (Dos Santos et al. 2000) or thioredoxin (encoded by dvu1839) (Valette et al. 2017) can be associated to general stress responses in NvH cells (Fig. 7).

### Further transformation of TP255 under oxic conditions

**Fig. 8:**
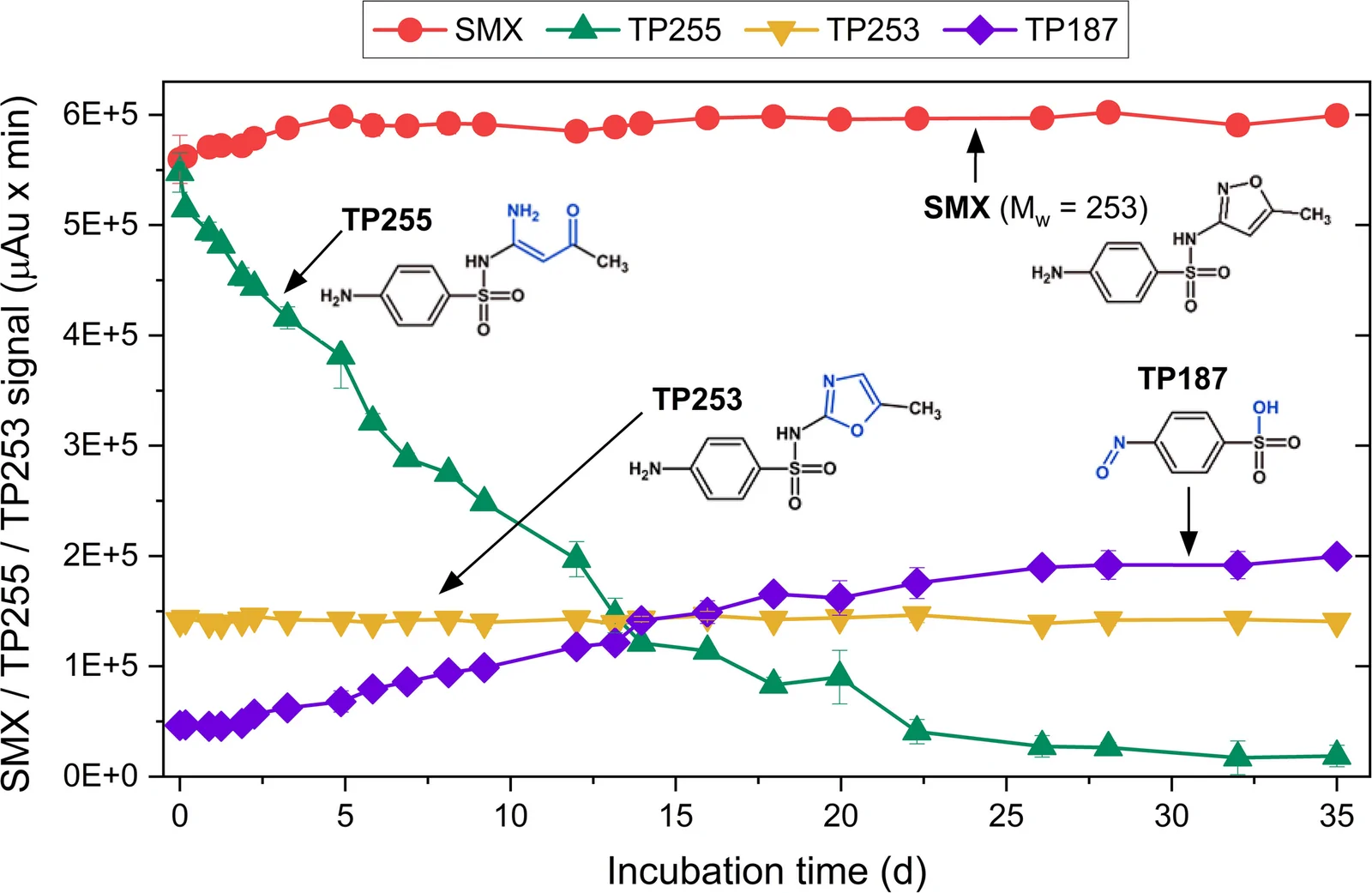
Abiotic oxic incubation of SMX, TP255, and TP253. Photometric absorption at 270 nm of 50 µM SMX (representing the residual, non-transformed SMX from the cultivation) and of TP255/TP253 in a 300-µL cell-free culture filtrate obtained from an anaerobic culture of NvH incubated with initially 100 µM SMX. The values of the predominant peak formed under oxic conditions referred to as TP187 are also shown. The shown chemical structures are inferred from their mass and their parent compounds while blue highlights regions of structural changes

In the anaerobic NvH cultures, TP255 and TP253 were stable over many months of incubation. However, when samples of the cultures were stored at +4 °C or −20 °C under oxic conditions, a slow decrease of TP255 over time was observed (data not shown). We demonstrated in a former study that anaerobically formed SMX TPs, including TP255 and TP253, underwent further aerobic microbial or oxic abiotic transformation (Akay et al. 2024). Here, we wanted to assess in more detail the oxic stability of TP255 and TP253 in filter-sterilized samples. We performed hourly sampling which allowed a kinetic description of abiotic transformation and it was also assessed if the TPs might spontaneously react back to SMX. During 35 days of incubation under oxic conditions, the signals of SMX and TP253 remained almost constant (Fig. 8) while the signal of TP255 decreased over time, noticeable already after 4 h of abiotic incubation. After 35 days, 97% of the TP255 signal had disappeared. Simultaneously, several other peaks with smaller intensities than SMX, TP255 and TP253, were observed at the UPLC-DAD (Fig. S7) with the largest peak (*m/z* = 188, TP 187) at a retention time (R_t_) of 4.44 min. Fragmentation analysis of TP187 using precursor ion scan and IDA triggered enhanced product ion (EPI) scan at LC-QTRAP-MS/MS (retention time = 2.0 min; *m/z* = 188) resulted in several main fragments with *m/z* values of 156, 108, 93, and 80 (Figs. S8, S9). These fragments and the UV absorption at 270 nm (Fig. S7B) suggested that TP187 contains the phenylsulfon aromatic ring system of SMX, but not the isoxazole ring. The difference in UPLC-DAD signal intensity between TP255 decrease and TP187 increase indicated that either TP255 was not stoichiometrically transformed into TP187 or TP187 has a lower absorption coefficient. Also, a minor amount of TP187 was already in the sample at the beginning of the incubation period. Abiotic transformation of TP255 followed first-order kinetics (R^2^ = 0.985; Fig. S10) and the first-order reaction constant *k*_*TP255*_ and environmental half-life (pH 8.0, room temperature) were 0.107 day^−1^ and 6.5 days, respectively.

## Discussion

### Cometabolic SMX transformation

The absence of considerable activity in controls (NCC, sulfide control) demonstrated that the observed SMX transformation was originating from the activity of the cells during anoxic batch cultivation (Fig. 4). Hence, we conclude that SMX transformation by NvH is a biologically mediated reductive process, which follows first-order reaction kinetics while sulfate is not limiting (Fig. 3a; Table S5). It leads to the transformation of SMX and the formation of two main TPs, TP255 and TP253 (Fig. 5). While TP255 is a reduced form of SMX, TP253 is an isomerized product of SMX with the isoxazole ring converted to an oxazole ring. Accordingly, for comparable cell densities (Fig. 5; Fig. S4) and similar SMX transforming activity per cell (Fig. S3), increasing electron donor concentrations promoted SMX transformation. We considered SMX adsorption to NvH cells negligible (Ouyang et al. 2021). The log *K*_*ow,SMX*_ is below 1 and the pH value during cultivation was between 7.0 and 7.2. Hence, SMX was in anionic state (*pK*_*a*2_ = 5.7) causing low tendency to adsorb to the negatively charged NvH cells. Previous experiments demonstrated negligible SMX adsorption to enriched bacterial cultures from sediment or sludge (Ouyang et al. 2021). Also, the dissipation of SMX coincided with the formation of TPs (e.g., Fig. 5) and therefore the decrease of SMX should primarily be due to biotransformation.

SMX was transformed without any considerable lag phase using a 3-day-old inoculum from SMX-acclimated but also from non-acclimated cells grown on lactate and sulfate (Fig. 2). Therefore, the presence of SMX seemed not to induce expression of enzymes specifically catalyzing SMX transformation. This conclusion was supported by our shotgun proteomics analysis comparing cells grown on H_2_-acetate-sulfate with and without SMX (Fig. 7). In these experiments, SMX did not induce a significant up-regulation of key catabolic proteins. Several protein candidates had been up- or down-regulated, but these were not considered key enzymes for energy conversation in NvH (Fig. 7) (Heidelberg et al. 2004; Keller and Wall 2011; Venceslau et al. 2014). It was striking that SMX transformation stopped or drastically slowed down once sulfate was depleted, which supports that SMX might have been cometabolically transformed by a reduced intermediate of the sulfate-reducing machinery. This could be proteins, such as dissimilatory sulfite reductase, the disulfide protein DsrC (Barbosa et al. 2024; Venceslau et al. 2014), a reduced ferredoxin or a cofactor. However, we have no indications from the proteomics data on the identity of such a protein or compound (Fig. 7). Also, our experiments cannot exclude metabolic SMX transformation, since minor growth or protein expression on the relatively low SMX concentrations might have evaded our detection. There are many unsolved questions in the understanding of the bioenergetics of sulfate reduction (Barbosa et al. 2024) and the reduction of SMX might be an interesting activity differentiating biochemical reduction activities.

The anaerobic conversion of SMX has its biotechnological relevance because SMX is often stable under oxic conditions and is thus regularly detected in the effluent of WWTPs (Kümmerer 2009a; Verlicchi et al. 2012a, b). Also, SMX can be detected and partially transformed in WWTPs sludge during digestion being exposed to sulfate-reducing and methanogenic conditions (Akay et al. 2024; Falås et al. 2016; Jia et al. 2017; Mohatt et al. 2011; Ouyang et al. 2021; Yang et al. 2018; Zhang et al. 2013). To also reduce the SMX load in WWTPs effluents, an anaerobic treatment of the wastewater mainstream could not only recover methane for energy (McCarty et al. 2011) but also allows for the transformation of SMX and other compounds that are amenable to primary anaerobic transformation (Akay et al. 2024). The toxicological relevance of the conversion of SMX to TP255 and TP253 needs more detailed investigation, but initial results have shown that the antibiotic effect is reduced (Ouyang et al. 2021).

### SMX transformation is coupled to lactate or H_2_ oxidation

The main reaction in the cultures was the oxidation of two moles of lactate to roughly two moles of acetate or the oxidation of four moles H_2_ with acetate as carbon source and the concomitant reduction of one mole of sulfate to sulfide. Therefore, it was possible to control the electron donor, electron acceptor, or carbon addition in a way that either lactate/H_2_ or sulfate was in excess in the culture.

We hypothesized that lactate and H_2_ oxidation is directly coupled to SMX transformation being enhanced by increasing lactate concentrations. This relationship persisted even when the culture entered and remained in the stationary phase for the majority of the incubation periods for lactate (day 4–149) (Figs. 5 and 6). Accordingly, once lactate was limiting but sulfate was still in excess (see 8 mM lactate culture), SMX transformation stalled after sulfide production (Fig. 5 and Fig. S4G). In cultures where lactate was in excess (24–40 mM) and sulfate was limiting, SMX was still transformed after sulfate was depleted but at a much slower rate.

SMX transformation only occurred in cultures with available electron donors and where active sulfate reduction took place at some point. Hence, one might conclude that enzymes that are responsible for lactate oxidation or sulfate reduction were still active on SMX after sulfate depletion (Fig. 5). Even though the SMX-Sul-H_2_-Ac cultures grew to higher cell densities and had higher sulfide production than the cultures grown on lactate, more SMX was transformed within the SMX-Sul-La cultures (Fig. 6). This suggested that lactate is not only the preferred physiological electron donor (Vita et al. 2015) but also that lactate stronger promotes the reduction of SMX than H_2_. Also, more of the transformed SMX was recovered as TP255 and TP253 (55–65%) using lactate as electron donor and carbon source versus hydrogen-acetate (27%). The estimated mass balances suggest the formation of unknow TPs or unsuitability of the same regression model for SMX and TP255/TP253.

### The anaerobically formed TP255 is unstable under oxic conditions

TP255, the major anaerobic TP formed by NvH from SMX, was unstable under oxic conditions and decomposed spontaneously in water almost completely within 35 days of incubation at 30 °C and pH 8.0 (Fig. 8). This suggests that a decomposition of SMX in wastewater would be possible during an anaerobic pre-treatment of SMX to TP255 and subsequent aerobic decomposition of TP255 during the activated sludge treatment (Akay et al. 2024). In contrast to TP255, both SMX and TP253 were stable under oxic conditions. This might be explained by their higher oxidation state and presence of the stable isoxazole/oxazole ring compared to TP255 (Zhang et al. 2023). The derived half-life of TP255 under oxic conditions was 6.5 days (*k*_*TP255*_ = 0.107 day^−1^, Fig. 8) and thus is more than sixfold faster than it took to biologically transform 50% of the 100 µM SMX by NvH in the presented study (ca. 40 days, Fig. 5). TP255 was not chemically altered at (de)protonating moieties; hence, we did not suspect its stability under oxic conditions to change as long as the pH value remains substantially above the *pK*_*a*2,SMX_ of 5.7. In the literature, different SMX half-lives were reported: 9–99 days for acclimated aerobic bacterial cultures (Baumgarten et al. 2011; Gauthier et al. 2010; Reis et al. 2014), 52 days for surface water (Prasannamedha And Kumar 2020), 15–18 days in soil samples under anaerobic conditions (Lin and Gan 2011), and 19 days for a large-scale surface water microcosm study (Lam et al. 2004). In comparison, the half-life of TP255 was remarkably shorter than that of SMX in the mentioned studies and also in this work. In consequence, conversion of SMX to TP255 by NvH leads to a lower persistency in the environment and better natural attenuation (Akay et al. 2024; Zhang et al. 2023). The LC-QTRAP-MS/MS mass spectra for TP187, the abiotic decomposition product of TP255, included the high-intensity fragments *m/z* 92, 108, and 156 (Fig. S10) and indicated an intact phenylsulfon structure (Akay et al. 2024). Despite that, the sulfonamide bond as bioactive center for SMX was cleaved, which may lead to diminished biological activity (Ouyang et al. 2021; Zhang et al. 2023) and weaker antibiotic resistance propagation compared to that of the mother compound SMX as suggested by Zhang et al. (2023).

## Conclusion

Overall, whole cells of NvH cometabolically and reductively transformed SMX coupling lactate or H_2_-acetate metabolism to sulfate reduction. SMX transformation was higher in younger cells and was influenced by the initial SMX concentration; however, it was independent of prior acclimation to SMX. The biologically formed TP255 (major TP) was instable in oxic atmosphere. This emphasizes the suitability of anaerobic pre-treatment (here: sulfate-reducing conditions) of micropollutant-contaminated waters, in order to render these recalcitrant water constituents more susceptible for a subsequent aerobic treatment (e.g., conventional activated sludge). Anaerobic pre-treatment and subsequent oxic conditions increase the natural attenuation potential of SMX and could also help to avoid the re-transformation of TPs back to the mother compound as reported in the literature. Hence, the contribution of SMX to antibiotic resistance propagation might be reduced. Overall, our findings highlight the potential of redox differentiation to promote natural attenuation of also other water contaminants. Further research should investigate SMX transforming proteins, the potential capability of other sulfate-reducing bacteria to transform SMX, and the potential capability of NvH to transform other sulfonamide antibiotics or micropollutants with an isoxazole ring diminishing their environmental persistence.

## Supplementary Information

Below is the link to the electronic supplementary material.

ESM 1 (XLSX 494 KB)

ESM 2 (DOCX 1.47 MB)

## Data Availability

The raw proteomic data has been deposited to the ProteomeXchange Consortium via the PRIDE partner repository with the dataset identifier PXD068774 and 10.6019/PXD068774.

## References

[CR1] Adler N, Balzer F, Blondzik K, Brauer F, Chorus I, Ebert I, Fiedler T, Grummt T, Heidemeier J, Hein A, Helmecke M, Hilliges F, Kirst I, Klasen J, Konradi S, Krause B, Küster A, Otto C, Pirntke U, Roskosch A, Schönfeld J, Selinka H-C, Szewzyl R, Westphal-Settele K, Straff W (2018) Antibiotika und Antibiotikaresistenzen in der Umwelt - Hintergrund, Herausforderungen und Handlungsoptionen. Umweltbundesamt (UBA), Dessau-Roßlau, p 44

[CR2] Adrian L, Manz W, Szewzyl U, Görisch H (1998) Physiological characterization of a bacterial consortium reductively dechlorinating 1,2,3- and 1,2,4-trichlorobenzene. Appl Environ Microbiol 64(2):496–503. https://doi.org/0099-2240/98/$04.00⫹0 9464384 10.1128/aem.64.2.496-503.1998 PMC106072

[CR3] Akay C, Ulrich N, Rocha U, Ding C, Adrian L (2024) Sequential anaerobic−aerobic treatment enhances sulfamethoxazole removal: from batch cultures to observations in a large-scale wastewater treatment plant. Environ Sci Technol. 10.1021/acs.est.4c00368 38973247 10.1021/acs.est.4c00368 PMC11256761

[CR4] Al Aukidy M, Verlicchi P, Jelic A, Petrovic M, Barcelo D (2012) Monitoring release of pharmaceutical compounds: occurrence and environmental risk assessment of two WWTP effluents and their receiving bodies in the Po Valley, Italy. Sci Total Environ 438:15–25. 10.1016/j.scitotenv.2012.08.061 22967493 10.1016/j.scitotenv.2012.08.061

[CR5] Alvarino T, Suarez S, Lema JM, Omil F (2014) Understanding the removal mechanisms of PPCPs and the influence of main technological parameters in anaerobic UASB and aerobic CAS reactors. J Hazard Mater 278:506–513. 10.1016/j.jhazmat.2014.06.031 25010455 10.1016/j.jhazmat.2014.06.031

[CR6] An J, Chen H, Wei S, Gu J (2015) Antibiotic contamination in animal manure, soil, and sewage sludge in Shenyang, northeast China. Environ Earth Sci 74(6):5077–5086. 10.1007/s12665-015-4528-y

[CR7] Aydin S, Ince B, Cetecioglu Z, Arikan O, Ozbayram EG, Shahi A, Ince O (2015) Combined effect of erythromycin, tetracycline and sulfamethoxazole on performance of anaerobic sequencing batch reactors. Bioresour Technol 186:207–214. 10.1016/j.biortech.2015.03.043 25817031 10.1016/j.biortech.2015.03.043

[CR8] Azimir N, Hassani AH, Darzi GN, Borghei SM (2017) Biodegradation of wastewater containing high concentration of Sulfamethoxazole by antibiotic adopted biofilm in attached growth bioreactors. Pol J Environ Stud 26(6):2463–2469

[CR9] Banin E, Hughes D, Kuipers OP (2017) Editorial: bacterial pathogens, antibiotics and antibiotic resistance. FEMS Microbiol Rev 41(3):450–452. 10.1093/femsre/fux016 28486583 10.1093/femsre/fux016

[CR10] Barbosa ACC, Venceslau SS, Pereira IAC (2024) DsrMKJOP is the terminal reductase complex in anaerobic sulfate respiration. Proc Natl Acad Sci U S A 121(6):e2313650121. 10.1073/pnas.2313650121 38285932 10.1073/pnas.2313650121 PMC10861901

[CR11] Baumgarten B, Jahrig J, Reemtsma T, Jekel M (2011) Long term laboratory column experiments to simulate bank filtration: factors controlling removal of sulfamethoxazole. Water Res 45(1):211–220. 10.1016/j.watres.2010.08.034 20828781 10.1016/j.watres.2010.08.034

[CR12] Ben W, Zhu B, Yuan X, Zhang Y, Yang M, Qiang Z (2018) Occurrence, removal and risk of organic micropollutants in wastewater treatment plants across China: comparison of wastewater treatment processes. Water Res 130:38–46. 10.1016/j.watres.2017.11.057 29197755 10.1016/j.watres.2017.11.057

[CR13] Benjamini Y, Hochberg Y (1995) Controlling the false discovery rate: a practical and powerful approach to multiple testing. J R Stat Soc 57(1):289–300

[CR14] Benotti MJ, Brownawell BJ (2009) Microbial degradation of pharmaceuticals in estuarine and coastal seawater. Environ Pollut 157(3):994–1002. 10.1016/j.envpol.2008.10.009 19038482 10.1016/j.envpol.2008.10.009

[CR15] Cabeza Y, Candela L, Ronen D, Teijon G (2012) Monitoring the occurrence of emerging contaminants in treated wastewater and groundwater between 2008 and 2010. The Baix Llobregat (Barcelona, Spain). J Hazard Mater 239:32–39. 10.1016/j.jhazmat.2012.07.032 22877748 10.1016/j.jhazmat.2012.07.032

[CR16] Carneiro RB, Sabatini CA, Santos-Neto AJ, Zaiat M (2019) Feasibility of anaerobic packed and structured-bed reactors for sulfamethoxazole and ciprofloxacin removal from domestic sewage. Sci Total Environ 678:419–429. 10.1016/j.scitotenv.2019.04.437 31077920 10.1016/j.scitotenv.2019.04.437

[CR17] Carvalho IT, Santos L (2016) Antibiotics in the aquatic environments: a review of the European scenario. Environ Int 94:736–757. 10.1016/j.envint.2016.06.025 27425630 10.1016/j.envint.2016.06.025

[CR18] Cetecioglu Z, Ince B, Azman S, Gokcek N, Coskun N, Ince O (2013) Determination of anaerobic and anoxic biodegradation capacity of sulfamethoxasole and the effects on mixed microbial culture biodegradation - Engineering and Technology

[CR19] Cetecioglu Z, Ince B, Orhon D, Ince O (2016) Anaerobic sulfamethoxazole degradation is driven by homoacetogenesis coupled with hydrogenotrophic methanogenesis. Water Res 90:79–89. 10.1016/j.watres.2015.12.013 26724442 10.1016/j.watres.2015.12.013

[CR20] Chatila S, Amparo MR, Carvalho LS, Penteado ED, Tomita IN, Santos-Neto AJ, Lima Gomes PC, Zaiat M (2016) Sulfamethoxazole and ciprofloxacin removal using a horizontal-flow anaerobic immobilized biomass reactor. Environ Technol 37(7):847–853. 10.1080/09593330.2015.1088072 26465824 10.1080/09593330.2015.1088072

[CR21] Chenxi W, Spongberg AL, Witter JD (2008) Determination of the persistence of pharmaceuticals in biosolids using liquid-chromatography tandem mass spectrometry. Chemosphere 73(4):511–518. 10.1016/j.chemosphere.2008.06.026 18674794 10.1016/j.chemosphere.2008.06.026

[CR22] Christou A, Aguera A, Bayona JM, Cytryn E, Fotopoulos V, Lambropoulou D, Manaia CM, Michael C, Revitt M, Schroder P, Fatta-Kassinos D (2017) The potential implications of reclaimed wastewater reuse for irrigation on the agricultural environment: the knowns and unknowns of the fate of antibiotics and antibiotic resistant bacteria and resistance genes - a review. Water Res 123:448–467. 10.1016/j.watres.2017.07.004 28689129 10.1016/j.watres.2017.07.004

[CR23] Cord-Ruwisch (1985) A quick method for the determ of diss and precip sulfides in cult of SRBs. J Microbiol Methods 4:33–36

[CR24] Ding C, Adrian L (2020) Comparative genomics in “*Candidatus Kuenenia stuttgartiensis*” reveal high genomic plasticity in the overall genome structure, CRISPR loci and surface proteins. BMC Genomics 21(1):851. 10.1186/s12864-020-07242-1 33261555 10.1186/s12864-020-07242-1 PMC7709395

[CR25] Dinh QT, Alliot F, Moreau-Guigon E, Eurin J, Chevreuil M, Labadie P (2011) Measurement of trace levels of antibiotics in river water using on-line enrichment and triple-quadrupole LC-MS/MS. Talanta 85(3):1238–1245. 10.1016/j.talanta.2011.05.013 21807177 10.1016/j.talanta.2011.05.013

[CR26] Dos Santos W, Pacheco I, Liu M-Y, Teixeira M, Xavier A, LeGall J (2000) Purification and characterization of an iron superoxide dismutase and a catalase from the sulfate-reducing bacterium *Desulfovibrio gigas*. J Bacteriol 182(3):796–804 10633116 10.1128/jb.182.3.796-804.2000 PMC94345

[CR27] Falås P, Wick A, Castronovo S, Habermacher J, Ternes TA, Joss A (2016) Tracing the limits of organic micropollutant removal in biological wastewater treatment. Water Res 95:240–249. 10.1016/j.watres.2016.03.009 26999256 10.1016/j.watres.2016.03.009 PMC5566204

[CR28] Fan CH, Yang CW, Chang BV (2019) Anaerobic degradation of sulfamethoxazole by mixed cultures from swine and sewage sludge. Environ Technol 40(2):210–218. 10.1080/09593330.2017.1384510 28942703 10.1080/09593330.2017.1384510

[CR29] Feng L, Casas ME, Ottosen LDM, Moller HB, Bester K (2017) Removal of antibiotics during the anaerobic digestion of pig manure. Sci Total Environ 603–604:219–225. 10.1016/j.scitotenv.2017.05.280 28628813 10.1016/j.scitotenv.2017.05.280

[CR30] Franklin AM, Williams CF, Andrews DM, Woodward EE, Watson JE (2016) Uptake of three antibiotics and an antiepileptic drug by wheat crops spray irrigated with wastewater treatment plant effluent. J Environ Qual 45(2):546–554. 10.2134/jeq2015.05.0257 27065402 10.2134/jeq2015.05.0257

[CR31] García Galán MJ, Diáz-Cruz MS, Barcelo D (2012) Removal of sulfonamide antibiotics upon conventional activated sludge and advanced membrane bioreactor treatment. Anal Bioanal Chem 404(5):1505–1515. 10.1007/s00216-012-6239-5 22825676 10.1007/s00216-012-6239-5

[CR32] Gartiser S, Urich E, Alexy R, Kümmerer K (2007) Ultimate biodegradation and elimination of antibiotics in inherent tests. Chemosphere 67(3):604–613. 10.1016/j.chemosphere.2006.08.038 17166562 10.1016/j.chemosphere.2006.08.038

[CR33] Gauthier H, Yargeau V, Cooper DG (2010) Biodegradation of pharmaceuticals by *Rhodococcus rhodochrous* and *Aspergillus niger* by co-metabolism. Sci Total Environ 408(7):1701–1706. 10.1016/j.scitotenv.2009.12.012 20089297 10.1016/j.scitotenv.2009.12.012

[CR34] Ghirardini A, Grillini V, Verlicchi P (2020) A review of the occurrence of selected micropollutants and microorganisms in different raw and treated manure - environmental risk due to antibiotics after application to soil. Sci Total Environ 707:136118. 10.1016/j.scitotenv.2019.136118 31881518 10.1016/j.scitotenv.2019.136118

[CR35] Goldstein M, Shenker M, Chefetz B (2014) Insights into the uptake processes of wastewater-borne pharmaceuticals by vegetables. Environ Sci Technol 48(10):5593–5600. 10.1021/es5008615 24749778 10.1021/es5008615

[CR36] Gros M, Rodriguez-Mozaz S, Barcelo D (2012) Fast and comprehensive multi-residue analysis of a broad range of human and veterinary pharmaceuticals and some of their metabolites in surface and treated waters by ultra-high-performance liquid chromatography coupled to quadrupole-linear ion trap tandem mass spectrometry. J Chromatogr A 1248:104–121. 10.1016/j.chroma.2012.05.084 22704668 10.1016/j.chroma.2012.05.084

[CR37] Grossberger A, Hadar Y, Borch T, Chefetz B (2014) Biodegradability of pharmaceutical compounds in agricultural soils irrigated with treated wastewater. Environ Pollut 185:168–177. 10.1016/j.envpol.2013.10.038 24286691 10.1016/j.envpol.2013.10.038

[CR38] Haveman SA, Greene EA, Stilwell CP, Voordouw JK, Voordouw G (2004) Physiological and gene expression analysis of inhibition of *Desulfovibrio vulgaris* hildenborough by nitrite. J Bacteriol 186(23):7944–7950. 10.1128/JB.186.23.7944-7950.2004 15547266 10.1128/JB.186.23.7944-7950.2004 PMC529081

[CR39] Heidelberg JF, Seshadri R, Haveman SA, Hemme CL, Paulsen IT, Kolonay JF, Eisen JA, Ward N, Methe B, Brinkac LM, Daugherty SC, Deboy RT, Dodson RJ, Durkin AS, Madupu R, Nelson WC, Sullivan SA, Fouts D, Haft DH, Selengut J, Peterson JD, Davidsen TM, Zafar N, Zhou L, Radune D, Dimitrov G, Hance M, Tran K, Khouri H, Gill J, Utterback TR, Feldblyum TV, Wall JD, Voordouw G, Fraser CM (2004) The genome sequence of the anaerobic, sulfate-reducing bacterium *Desulfovibrio vulgaris* Hildenborough. Nat Biotechnol 22(5):554–559. 10.1038/nbt959 15077118 10.1038/nbt959

[CR40] Homem V, Santos L (2011) Degradation and removal methods of antibiotics from aqueous matrices - a review. J Environ Manage 92(10):2304–2347. 10.1016/j.jenvman.2011.05.023 21680081 10.1016/j.jenvman.2011.05.023

[CR41] Hu X, Zhou Q, Luo Y (2010) Occurrence and source analysis of typical veterinary antibiotics in manure, soil, vegetables and groundwater from organic vegetable bases, northern China. Environ Pollut 158(9):2992–2998. 10.1016/j.envpol.2010.05.023 20580472 10.1016/j.envpol.2010.05.023

[CR42] Huang X, Ye ZL, Cai J, Lin L (2021) Quantification of DOM effects on tetracyclines transport during struvite recovery from swine wastewater. Water Res 206:117756. 10.1016/j.watres.2021.117756 34678697 10.1016/j.watres.2021.117756

[CR43] Huber MM, Göbel A, Joss A, Hermann N, Löffler D, Mcardel CS, Ried A, Siegrist H, Ternes T, Von Gunten U (2005) Oxidation of pharmaceuticals during ozonation of municipal wastewater effluents: a pilot study. Environ Sci Technol 39:4290–4299 15984812 10.1021/es048396s

[CR44] Jia Y, Khanal SK, Zhang H, Chen GH, Lu H (2017) Sulfamethoxazole degradation in anaerobic sulfate-reducing bacteria sludge system. Water Res 119:12–20. 10.1016/j.watres.2017.04.040 28433879 10.1016/j.watres.2017.04.040

[CR45] Joss A, Zabczynski S, Gobel A, Hoffmann B, Loffler D, McArdell CS, Ternes TA, Thomsen A, Siegrist H (2006) Biological degradation of pharmaceuticals in municipal wastewater treatment: proposing a classification scheme. Water Res 40(8):1686–1696. 10.1016/j.watres.2006.02.014 16620900 10.1016/j.watres.2006.02.014

[CR46] Keller KL, Wall JD (2011) Genetics and molecular biology of the electron flow for sulfate respiration in desulfovibrio. Front Microbiol 2:135. 10.3389/fmicb.2011.00135 21747813 10.3389/fmicb.2011.00135 PMC3129016

[CR47] Kibuye FA, Gall HE, Elkin KR, Swistock B, Veith TL, Watson JE, Elliott HA (2019) Occurrence, concentrations, and risks of pharmaceutical compounds in private wells in central Pennsylvania. J Environ Qual 48(4):1057–1066. 10.2134/jeq2018.08.0301 31589682 10.2134/jeq2018.08.0301

[CR48] Klein EY, Van Boeckel TP, Martinez EM, Pant S, Gandra S, Levin SA, Goossens H, Laxminarayan R (2018) Global increase and geographic convergence in antibiotic consumption between 2000 and 2015. Proc Natl Acad Sci U S A 115(15):E3463–E3470. 10.1073/pnas.1717295115 29581252 10.1073/pnas.1717295115 PMC5899442

[CR49] Kolpin DW, Furlong ET, Meyer MT, Thurman EM, Zaugg SD, Barber LB, Buxton HT (2002) Pharmaceuticals, hormones, and other organic wastewater contaminants in U.S. streams, 1999–2000: a national reconnaissance. Microorganisms 7:1–24. 10.1021/es011055j 10.1021/es011055j 11944670

[CR50] Kraemer SA, Ramachandran A, Perron GG (2019) Antibiotic pollution in the environment: from microbial ecology to public policy. Microorganisms. 10.3390/microorganisms7060180 31234491 10.3390/microorganisms7060180 PMC6616856

[CR51] Kümmerer K (2009a) Antibiotics in the aquatic environment–a review–part I. Chemosphere 75(4):417–434. 10.1016/j.chemosphere.2008.11.086 19185900 10.1016/j.chemosphere.2008.11.086

[CR52] Kümmerer K (2009b) Antibiotics in the aquatic environment-a review-part II. Chemosphere 75(4):435–441. 10.1016/j.chemosphere.2008.12.006 19178931 10.1016/j.chemosphere.2008.12.006

[CR53] Lam MW, Young CJ, Brain RA, Johnson DJ, Hanson MA (2004) Aquatic persistence of eight pharmaceuticals in a microcosm study. Environ Toxicol Chem 23(6):1431–1440 15376529 10.1897/03-421

[CR54] Li B, Zhang T (2010) Biodegradation and adsorption of antibiotics in the activated sludge process. Environ Sci Technol 44:3468–3473 20384353 10.1021/es903490h

[CR55] Lin K, Gan J (2011) Sorption and degradation of wastewater-associated non-steroidal anti-inflammatory drugs and antibiotics in soils. Chemosphere 83(3):240–246. 10.1016/j.chemosphere.2010.12.083 21247615 10.1016/j.chemosphere.2010.12.083

[CR56] Lovley DR, Phillips EJP (1994) Redcution of chromate by *Desulfovibrio vulgaris* and its *c*_*3*_ cytochrome. Appl Environ Microbiol 60:726–728 16349200 10.1128/aem.60.2.726-728.1994 PMC201373

[CR57] Lovley DR, Phillips EJP, Woodward JC (1993a) Enzymatic iron and uranium reduction by sulfate reducing bacteria. Mar Geol 113:41–53

[CR58] Lovley DR, Widman PK, Woodward JC, Phillips EJP (1993b) Reduction of uranium by cytochrome *c*_*3*_ of *Desulfovibrio vulgaris*. Appl Environ Microbiol 59(11):3572–3576 8285665 10.1128/aem.59.11.3572-3576.1993 PMC182500

[CR59] Malchi T, Maor Y, Tadmor G, Shenker M, Chefetz B (2014) Irrigation of root vegetables with treated wastewater: evaluating uptake of pharmaceuticals and the associated human health risks. Environ Sci Technol 48(16):9325–9333. 10.1021/es5017894 25026038 10.1021/es5017894

[CR60] McCarty PL, Bae J, Kim J (2011) Domestic wastewater treatment as a net energy producer–can this be achieved? Environ Sci Technol 45(17):7100–7106. 10.1021/es2014264 21749111 10.1021/es2014264

[CR61] Mohatt JL, Hu L, Finneran KT, Strathmann TJ (2011) Microbially mediated abiotic transformation of the antimicrobial agent sulfamethoxazole under iron-reducing soil conditions. Environ Sci Technol 45(11):4793–4801. 10.1021/es200413g 21542626 10.1021/es200413g

[CR62] Mohring SAI, Strzych I, Fernandes MR, Kiffmeyer TK, Tuerk J, Hamscher G (2009) Degradation end elimination of various sulfonamides during anaerobic fermentation. Environ Sci Technol 43(7):2569–2574. 10.1021/es802042d 19452918 10.1021/es802042d

[CR63] Morasch B, Bonvin F, Reiser H, Grandjean D, de Alencastro f, Perazzolo C, Chèvere N, Kohn T (2010) Occurrence and fate of micropollutants in the Vidy Bay of Lake Geneva, Switzerland. Part II: micropollutant removal between wastewater and raw drinking water. Environ Toxicol Chem. 10.1002/etc.222 20821617 10.1002/etc.222

[CR64] Nödler K, Licha T, Barbieri M, Perez S (2012) Evidence for the microbially mediated abiotic formation of reversible and non-reversible sulfamethoxazole transformation products during denitrification. Water Res 46(7):2131–2139. 10.1016/j.watres.2012.01.028 22326197 10.1016/j.watres.2012.01.028

[CR65] O´Neill J (2014) Antimicrobial resistance: tackling a crisis for the health and wealth of nations. Wellcome Trust, London. https://wellcomecollection.org/works/rdpck35v

[CR66] Odom JM, Peck HD (1981) Hydrogen cycling as a general mechanism for energy coupling in the sulfate-reducing bacteria, Desulfovibrio sp. FEMS Microbiol Lett 12(1):47–50. https://www.sciencedirect.com/science/article/pii/0378109781900355

[CR67] Onesios KM, Yu JT, Bouwer EJ (2009) Biodegradation and removal of pharmaceuticals and personal care products in treatment systems: a review. Biodegradation 20(4):441–466. 10.1007/s10532-008-9237-8 19112598 10.1007/s10532-008-9237-8

[CR68] Ouyang WY, Su JQ, Richnow HH, Adrian L (2019) Identification of dominant sulfamethoxazole-degraders in pig farm-impacted soil by DNA and protein stable isotope probing. Environ Int 126:118–126. 10.1016/j.envint.2019.02.001 30797101 10.1016/j.envint.2019.02.001

[CR69] Ouyang WY, Birkigt J, Richnow HH, Adrian L (2021) Anaerobic transformation and detoxification of sulfamethoxazole by sulfate-reducing enrichments and *Desulfovibrio vulgaris*. Environ Sci Technol 55(1):271–282. 10.1021/acs.est.0c03407 33350822 10.1021/acs.est.0c03407

[CR70] Padhye LP, Yao H, Kung’u FT, Huang CH (2014) Year-long evaluation on the occurrence and fate of pharmaceuticals, personal care products, and endocrine disrupting chemicals in an urban drinking water treatment plant. Water Res 51:266–276. 10.1016/j.watres.2013.10.070 24262763 10.1016/j.watres.2013.10.070

[CR71] Pereira PM, He Q, Valente FM, Xavier AV, Zhou J, Pereira IA, Louro RO (2008) Energy metabolism in *Desulfovibrio vulgaris* Hildenborough: insights from transcriptome analysis. Antonie Van Leeuwenhoek 93(4):347–362. 10.1007/s10482-007-9212-0 18060515 10.1007/s10482-007-9212-0

[CR72] Pereira IA, Ramos AR, Grein F, Marques MC, da Silva SM, Venceslau SS (2011) A comparative genomic analysis of energy metabolism in sulfate reducing bacteria and archaea. Front Microbiol 2:69. 10.3389/fmicb.2011.00069 21747791 10.3389/fmicb.2011.00069 PMC3119410

[CR73] Pieulle L, Morelli X, Gallice P, Lojou E, Barbier P, Czjzek M, Bianco P, Guerlesquin F, Hatchikian EC (2005) The type I/type II cytochrome c3 complex: an electron transfer link in the hydrogen-sulfate reduction pathway. J Mol Biol 354(1):73–90. 10.1016/j.jmb.2005.09.036 16226767 10.1016/j.jmb.2005.09.036

[CR74] Pires RH, Lourenco AI, Morais F, Teixeira M, Xavier AV, Saraiva LM, Pereira IA (2003) A novel membrane-bound respiratory complex from *Desulfovibrio desulfuricans* ATCC 27774. Biochim Biophys Acta 1605(1–3):67–82. 10.1016/s0005-2728(03)00065-3 12907302 10.1016/s0005-2728(03)00065-3

[CR75] Pires RH, Venceslau SS, Morais F, Teixeira M, Xavier AV, Pereira IA (2006) Characterization of the *Desulfovibrio desulfuricans* ATCC 27774 DsrMKJOP complex - a membrane-bound redox complex involved in the sulfate respiratory pathway. Biochemistry 45:249–262. 10.1021/bi0515265 16388601 10.1021/bi0515265

[CR76] Prasannamedha G, Kumar PS (2020) A review on contamination and removal of sulfamethoxazole from aqueous solution using cleaner techniques: present and future perspective. Journal of Cleaner Production 250:119553. 10.1016/j.jclepro.2019.119553

[CR77] Radke M, Lauwigi C, Heinkele G, Mürdter TEM, Letzel M (2009) Fate of the antibiotic sulfamethoxazole and its two major human metabolites in a water sediment test. Environ Sci Technol 43:3135–3141 19534125 10.1021/es900300u

[CR78] Reis PJ, Reis AC, Ricken B, Kolvenbach BA, Manaia CM, Corvini PF, Nunes OC (2014) Biodegradation of sulfamethoxazole and other sulfonamides by *Achromobacter denitrificans* PR1. J Hazard Mater 280:741–749. 10.1016/j.jhazmat.2014.08.039 25238191 10.1016/j.jhazmat.2014.08.039

[CR79] Rizzo L, Manaia C, Merlin C, Schwartz T, Dagot C, Ploy MC, Michael I, Fatta-Kassinos D (2013) Urban wastewater treatment plants as hotspots for antibiotic resistant bacteria and genes spread into the environment: a review. Sci Total Environ 447:345–360. 10.1016/j.scitotenv.2013.01.032 23396083 10.1016/j.scitotenv.2013.01.032

[CR80] Rosenblatt-Farrell N (2009) The landscape of antibiotic resistance. Environ Health Perspect. 10.1289/ehp.117-a244 19590668 10.1289/ehp.117-a244 PMC2702430

[CR81] Tang Y, Pingitore F, Mukhopadhyay A, Phan R, Hazen TC, Keasling JD (2007) Pathway confirmation and flux analysis of central metabolic pathways in *Desulfovibrio vulgaris* hildenborough using gas chromatography-mass spectrometry and Fourier transform-ion cyclotron resonance mass spectrometry. J Bacteriol 189(3):940–949. 10.1128/JB.00948-06 17114264 10.1128/JB.00948-06 PMC1797301

[CR82] Valette O, Tran TTT, Cavazza C, Caudeville E, Brasseur G, Dolla A, Talla E, Pieulle L (2017) Biochemical function, molecular structure and evolution of an atypical thioredoxin reductase from *Desulfovibrio vulgaris*. Front Microbiol 8:1855. 10.3389/fmicb.2017.01855 29033913 10.3389/fmicb.2017.01855 PMC5627308

[CR83] Van Boeckel TP, Gandra S, Ashok A, Caudron Q, Grenfell BT, Levin SA, Laxminarayan R (2014) Global antibiotic consumption 2000 to 2010: an analysis of national pharmaceutical sales data. Lancet Infect Dis 14(8):742–750. 10.1016/S1473-3099(14)70780-7 25022435 10.1016/S1473-3099(14)70780-7

[CR84] van Hoek AH, Mevius D, Guerra B, Mullany P, Roberts AP, Aarts HJ (2011) Acquired antibiotic resistance genes: an overview. Front Microbiol 2:203. 10.3389/fmicb.2011.00203 22046172 10.3389/fmicb.2011.00203 PMC3202223

[CR85] Venceslau SS, Stockdreher Y, Dahl C, Pereira IA (2014) The “bacterial heterodisulfide” DsrC is a key protein in dissimilatory sulfur metabolism. Biochim Biophys Acta 1837(7):1148–1164. 10.1016/j.bbabio.2014.03.007 24662917 10.1016/j.bbabio.2014.03.007

[CR86] Verlicchi P, Al Aukidy M, Galletti A, Petrovic M, Barcelo D (2012a) Hospital effluent: investigation of the concentrations and distribution of pharmaceuticals and environmental risk assessment. Sci Total Environ 430:109–118. 10.1016/j.scitotenv.2012.04.055 22634557 10.1016/j.scitotenv.2012.04.055

[CR87] Verlicchi P, Al Aukidy M, Zambello E (2012b) Occurrence of pharmaceutical compounds in urban wastewater: removal, mass load and environmental risk after a secondary treatment–a review. Sci Total Environ 429:123–155. 10.1016/j.scitotenv.2012.04.028 22583809 10.1016/j.scitotenv.2012.04.028

[CR88] Vita N, Valette O, Brasseur G, Lignon S, Denis Y, Ansaldi M, Dolla A, Pieulle L (2015) The primary pathway for lactate oxidation in *Desulfovibrio vulgaris*. Front Microbiol 6:606. 10.3389/fmicb.2015.00606 26167158 10.3389/fmicb.2015.00606 PMC4481167

[CR89] Voordouw G (2002) Carbon monoxide cycling by *Desulfovibrio vulgaris* Hildenborough. J Bacteriol 184(21):5903–5911. 10.1128/JB.184.21.5903-5911.2002 12374824 10.1128/JB.184.21.5903-5911.2002 PMC135394

[CR90] Walker CB, He Z, Yang ZK, Ringbauer JA Jr., He Q, Zhou J, Voordouw G, Wall JD, Arkin AP, Hazen TC, Stolyar S, Stahl DA (2009) The electron transfer system of syntrophically grown *Desulfovibrio vulgaris*. J Bacteriol 191(18):5793–5801. 10.1128/JB.00356-09 19581361 10.1128/JB.00356-09 PMC2737945

[CR91] Wang J, Wang S (2018) Microbial degradation of sulfamethoxazole in the environment. Appl Microbiol Biotechnol 102(8):3573–3582. 10.1007/s00253-018-8845-4 29516143 10.1007/s00253-018-8845-4

[CR92] WHO (2017) Guidelines for drinking-water quality: fourth edition incorporating the first addendum. Geneva: World Health Organization, New York. https://creativecommons.org/licenses/by-nc-sa/3.0/igo 28759192

[CR93] Xu W, Zhang G, Li X, Zou S, Li P, Hu Z, Li J (2007) Occurrence and elimination of antibiotics at four sewage treatment plants in the Pearl River Delta (PRD), South China. Water Res 41(19):4526–4534. 10.1016/j.watres.2007.06.023 17631935 10.1016/j.watres.2007.06.023

[CR94] Yang SF, Lin CF, Lin AY, Hong PK (2011) Sorption and biodegradation of sulfonamide antibiotics by activated sludge: experimental assessment using batch data obtained under aerobic conditions. Water Res 45(11):3389–3397. 10.1016/j.watres.2011.03.052 21529876 10.1016/j.watres.2011.03.052

[CR95] Yang CW, Tsai LL, Chang BV (2018) Anaerobic degradation of sulfamethoxazole in mangrove sediments. Sci Total Environ 643:1446–1455. 10.1016/j.scitotenv.2018.06.305 30189561 10.1016/j.scitotenv.2018.06.305

[CR96] Yu TH, Lin AY, Panchangam SC, Hong PK, Yang PY, Lin CF (2011) Biodegradation and bio-sorption of antibiotics and non-steroidal anti-inflammatory drugs using immobilized cell process. Chemosphere 84(9):1216–1222. 10.1016/j.chemosphere.2011.05.045 21684572 10.1016/j.chemosphere.2011.05.045

[CR97] Zaman SB, Hussain MA, Nye R, Mehta V, Mamun KT, Hossain N (2017) A review on antibiotic resistance: alarm bells are ringing. Cureus 9(6):e1403. 10.7759/cureus.1403 28852600 10.7759/cureus.1403 PMC5573035

[CR98] Zhang Y, Xu J, Zhong Z, Guo C, Li L, He Y, Fan W, Chen Y (2013) Degradation of sulfonamides antibiotics in lake water and sediment. Environ Sci Pollut Res Int 20(4):2372–2380. 10.1007/s11356-012-1121-8 22903812 10.1007/s11356-012-1121-8

[CR99] Zhang QQ, Ying GG, Pan CG, Liu YS, Zhao JL (2015) Comprehensive evaluation of antibiotics emission and fate in the river basins of China: source analysis, multimedia modeling, and linkage to bacterial resistance. Environ Sci Technol 49(11):6772–6782. 10.1021/acs.est.5b00729 25961663 10.1021/acs.est.5b00729

[CR100] Zhang H, Quan H, Song S, Sun L, Lu H (2023) Comprehensive assessment of toxicity and environmental risk associated with sulfamethoxazole biodegradation in sulfur-mediated biological wastewater treatment. Water Res 246:120753. 10.1016/j.watres.2023.120753 37871376 10.1016/j.watres.2023.120753

