# Supplementary material for: Physiological characterization of anaerobic cometabolic transformation of sulfamethoxazole by Nitratidesulfovibrio vulgaris Hildenborough
Source: Appl Microbiol Biotechnol. 2026 Mar 31;110(1):129. doi: 10.1007/s00253-026-13792-3 (PMC13043603; doi:10.1007/s00253-026-13792-3)
Supplement: Supplementary file 2 — (DOCX 1.47 MB) [file 253_2026_13792_MOESM2_ESM.docx]

# Physiological characterization of anaerobic cometabolic transformation of sulfamethoxazole by *Nitratidesulfovibrio vulgaris* Hildenborough

Jimmy Köpke^1,2,3^, Wei-Ying Ouyang^1^, Caglar Akay^1^, Chang Ding^1,*^, Aki Sebastian Ruhl^2,3^, Lorenz Adrian^1,4^

^1^Department Molecular Environmental Biotechnology, Helmholtz Centre for Environmental Research - UFZ, Leipzig, Germany

^2^German Environment Agency, Section II 3.3, Berlin, Germany

^3^Chair of Water Treatment, Technische Universität Berlin KF4, Berlin, Germany

^4^Chair of Geobiotechnology, Technische Universität Berlin, Berlin, Germany

*corresponding author: Chang Ding, [chang.ding@ufz.de](mailto:chang.ding@ufz.de); ORCID: 0000-0001-5550-4685

Journal: Applied Microbiology and Biotechnology

Supplemental Information

## Supplementary Methods

### Text S1: Preparation of anaerobic medium for Nitratidesulfovibrio vulgaris Hildenborough

A defined mineral culture medium was used for cultivation composed of salts (200 mg L^-1^ K_2_HPO_4_, 270 mg L^-1^ NH_4_Cl, 1000 mg L^-1^ NaCl, 410 mg L^-1^ MgCl_2_ × 6H_2_O, 520 mg L^-1^ KCl, and 150 mg L^-1^ CaCl_2_ × 2H_2_O), 1 mL/L SL-9 trace element solution (2 mg L^-1^ FeCl_2_ × 4H_2_O, 12.8 mg L^-1^ Nitriloacetic acid, 0.19 mg L^-1^ CoCl_2_ × 6H_2_O, 0.08 mg L^-1^ MnCl_2_ × 2H_2_O, 0.07 mg L^-1^ ZnCl_2_, 0.036 mg L^-1^ Na_2_MoO_4_ × 2H_2_O, 0.024 mg L^-1^ NiCl_2_ × 6H_2_O, 0.006 mg L^-1^ H_3_BO_3_ and 0.002 mg L^-1^ CuCl_2_ × 2H_2_O), 1 mL/L Se/W solution (500 mg L^-1^ NaOH, 6 mg L^-1^ Na_2_SeO_3_ × 5H_2_O and 8 mg L^-1^ Na_2_WO_4_ × 2H_2_O) and Na-resazurin as a redox indicator. The medium was flushed with N2 for 60 min and distributed into serum bottles within an anaerobic glove box (Coy Laboratory Inc., USA) with a gas phase of 97% N_2_ and 3% H_2_. The bottles were then crimped with butyl rubber septa and aluminum caps and subsequently autoclaved (121°C, 40 min). After cooling, autoclaved NaHCO_3_ (10 mM) as pH buffer, sterile-filtered L-cysteine as reducing agent (2 mM) and sterile-filtered vitamins (20 mg L^-1^ biotin, 20 mg L^-1^ folic acid, 252 mg L^-1^ pyridoxine-HCl, 50 mg L^-1^ riboflavin, 50 mg L^-1^ thiamine-HCl, 50 mg L^-1^ nicotinic acid, 50 mg L^-1^ Ca-D-pantothenate, 50 mg L^-1^ p-Aminobenzoic acid, 50 mg L^-1^ vitamin B12 and 50 mg L^-1^ α-Lipoic acid) were added to each bottle using sterile 1 mL syringes and needles. Lactate as electron donor and sulfate as electron acceptor were amended using sterile-filtered (0.2 µm CA syringe filter) stock solutions of 4 M Na-(L)-lactate and 500 mM K_2_SO_4_ at varying final concentrations. If not stated otherwise, all used solutions were anaerobic and sterile. All bottles were incubated overnight at 30°C in the dark without shaking before inoculation and stored at room temperature until used for cultivation of *Nitratidesulfovibrio vulgaris* Hildenborough (NvH).

**Table S1** Composition of mineral salts solution (50x) in 1 liter (Widdel 1980). The solution was sterilized by autoclaving at 121°C for 40 min.

| Salt | Amount |
| --- | --- |
| KH_2_PO_4_ | 10 g |
| NH_4_Cl | 13.5 g |
| NaCl | 50 g |
| MgCl_2_ x 6 H_2_O | 20.5 g |
| KCl | 26 g |
| CaCl_2_ x 2 H_2_O | 7.5 g |

**Table S2** Composition of the trace elements solution SL9 (1000x) in 1 liter as by Adrian et al. (1998). The pH was adjusted to 7.0 using NaOH and the solution sterilized by autoclaving at 121°C for 20 min.

| Salt | Amount |
| --- | --- |
| FeCl_2_ | 2 g |
| Nitrilotriacetic acid (NTA) | 12.8 g |
| CoCl_2_ x 6H_2_O | 190 mg |
| MnCl_2_ x 2H_2_O | 80 mg |
| ZnCl_2_ | 70 mg |
| Na_2_MoO_4_ x 2H_2_O | 36 mg |
| NiCl_2_ x 6H_2_O | 24 mg |
| H_3_BO_6_ | 6 mg |
| CuCl_2_ x 2H_2_O | 2 mg |

**Note**: The chemicals were dissolved separately in small volumes of H_2_O, except FeCl_2_ which was dissolved in 300-500 mL H_2_O with nitrilotriacetic acid (NTA; pH 7.0).

**Table S3** Composition of the Vitamin 10 stock solution (1000x) in 1 liter water. The solutions pH was adjusted to pH 7.0 using 5 M NaOH and subsequently sterile filtrated and kept at 4°C in the dark.

| Vitamin | Amount |
| --- | --- |
| D(+)Biotin | 20 mg |
| Folic acid | 20 mg |
| Pyridoxamine dihydrochloride monohydrate | 252 mg |
| (-)-Riboflavin | 50 mg |
| Thiamine chloride hydrochloride | 50 mg |
| Nicotine acid | 50 mg |
| Calcium-D(+)-pantothenate | 50 mg |
| p-aminobenzoic acid | 50 mg |
| DL-α-Lipoic acid | 50 mg |
| Vitamin B12 | 50 mg |

**Table S4** Composition of reaction mix for whole-cell *in-vitro* activity test for SMX transformation by NvH.

| **Ingredients** | **Final concentration** |
| --- | --- |
| Anoxic sterile water | Fill up to final volume |
| Potassium phosphate buffer (1M, pH 6.5) | 200 mM |
| Methyl viologen | 1 mM |
| Ti(III)citrate | 1 mM |
| SMX | 100 µM |

### Text S2: Cultural growth monitoring

To monitor the cultural growth, 20 µL of culture sample were amended with 2.6 µL SYBR-Green staining dye and incubated for 10 min at room temperature in the dark. Afterwards, 18 µL of the stained samples were transferred onto an agarose-coated microscopy slide and cell density was estimated (cells mL^-1^) using a Nikon OptiPhat-2 phase contrast microscope with an attached LED lamp. With an excitation at 490 nm, stained DNA within the cells emitted green light and at minimum 15 pictures for each sample were taken. All steps were executed in the dark in order to protect the light-sensitive staining dye from light. Light signals were automatically counted via ImageJ macros and averaged. Alternatively, to epi fluorescence microscopy, the growth of NvH was estimated based on the optical density at 600 nm (OD_600_) for a 1 mL sample using a spectrophotometer.

### Text S3: Ion source-dependent MS parameters at LC-MS/MS-QTRAP

The ion source-dependent MS parameters for identification and structure verification of SMX and known TPs were as follows: Curtain gas pressure = 35 psi; ion spray (IS) voltage = +5000 V; turbo spray temperature = 450°C; nebulizer gas pressure = 60 psi; heater gas pressure = 60 psi; collision-induced dissociation gas = medium. Nitrogen was used as curtain and collision gas. The compound-dependent MS parameters declustering potential, entrance potential, collision energy and collision cell exit potential were optimized by direct infusion of standard solutions of SMX at concentrations ranging from 10 to 20 μg L^-1^.

### Text S4: Comparative shot-gun proteomics

Protein crude extract was obtained from a 30°C grown culture of NvH harvested at day 31 of incubation at stationary phase by centrifugation at 10,000 g at 4°C. Supernatant was removed and 30 µL ammonium bicarbonate buffer (ammonium bicarbonate, 100 mM) added to each sample. Three cycles of freeze/thaw (- 80°C / 40°C) were used to disrupt the cell pellet. In order to account for sample to sample variation in protein content, 100 ng BSA as internal standard was spiked to each sample. Afterwards, 5% (v/v) sodium deoxycholate for better unfolding of the proteins, 12 mM dithiothreitol (DDT) as reducing agent for the disulfide bond of the peptide sulfur groups, and 40 mM of 2-iodacetamide (IAA) to protect the reduced cysteine SH-groups from re-oxidation were added. After vortexing and incubation at 37°C for 30 min at 400 rpm, 100 mM ammonium bicarbonate buffer was added and thereby the DOC diluted to 1%. The protein extract was tryptically digested overnight at 37°C and 400 rpm using 0.5 µg reductively methylated trypsin (Promega, USA). Next day, digestion was stopped by adding 2% (v/v) of neat forming acid to each vial. Each sample was vortexed vigorously to homogenize, undigested proteins and precipitated DOC were removed via two rounds of centrifugation at 16,100 g for 10 min. The digested peptide was desalted by using 100-µL C_18_ ziptips (Pierce^TM^, Thermo Fisher Scientific, USA) as previously described in Ding and Adrian (2020). The desalted peptides were vacuum dried and stored at -20°C until analysis. Before analysis, dried peptides were resuspended in 55 µL 0.1 % formic acid (MS-grade), shaken vigorously for 10 min, briefly ultrasonicated and centrifuged at 16,100 g for 10 min. The supernatant (3 µL) was analyzed by nLC-Orbitrap as previously described (Ding and Adrian, 2020). Prior to analysis, the peptide concentration was determined based on the absorption at 205 nm on a spectrophotometer (Denovix DS-11, USA) using 0.1 % FA as blank.

Protein identification and statistical analysis was carried out using Proteome Discoverer 2.4 (Thermo Fisher Scientific, USA). Precursor abundance was calculated based on intensity, and protein abundance calculated from summed peptide abundances. Protein abundance ratios were calculated based on pair-wise comparison of each replicate among triplicates of each culture. Maximum allowed protein fold-changes were set to 100 and missing peptide signals in some of the triplicates were imputated via replicate-based resampling (random values sampled from distributions around the medians of detected values of replicates). Statistical significance was calculated using a background-based t-test with Benajmini-Hochberg correction and the adjusted significance thresholds were set to 0.05.

## Supplementary Figures and Tables

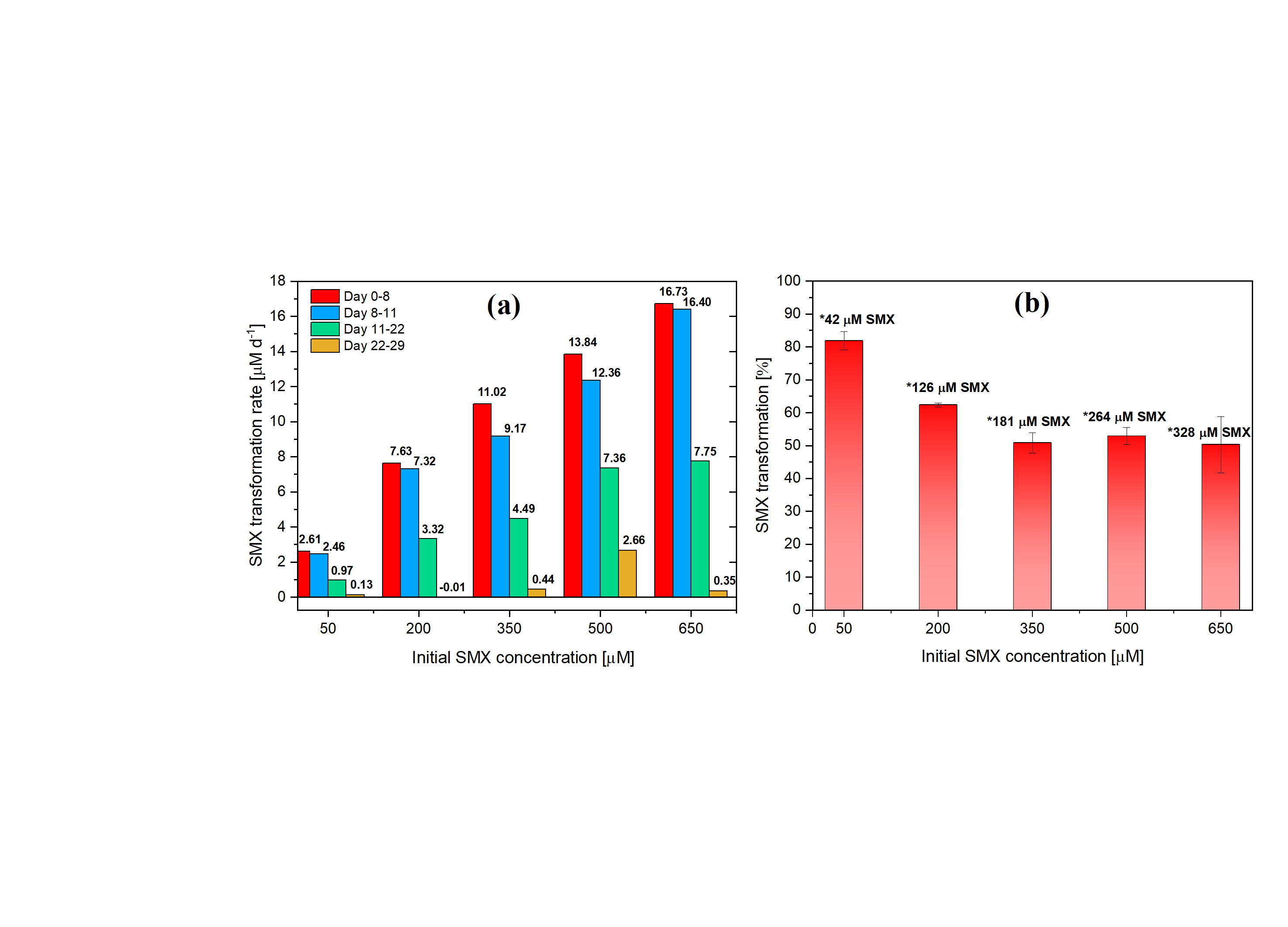
**Fig. S1** Sulfate-reducing activity and growth in cultures of *N. vulgaris* with 100 µM SMX inoculated with cells from the stationary phase (14 days culture age). Shown are sulfate concentrations (a), sulfide concentrations (b) and cell growth (c). Cultures contained 100 µM SMX, 17.8 mM lactate and 20 mM K_2_SO_4_. Also included is a No Cell Control (NCC), a “No SMX Control" (NSC) and a “Sulfide Control” which only contained 100 µM SMX and 8 mM Na_2_S to test for abiotic conversion.

**Fig. S2** SMX transformation by *N. vulgaris* with 50–650 µM initial SMX concentration. **(a)** SMX transformation rates [µM d^-1^] for the various initial SMX concentrations between the sampling points Day 0–8, Day 8–11, Day 11–22, and Day 22–29. **(b)** SMX transformation [%] for different initial SMX concentrations after 28 days of incubation showing the average total transformed amount of SMX [µM] indicated by “*”.

**Fig. S3** Relative SMX transformation by *N. vulgaris* with 50–650 µM initial SMX concentration and constant lactate und sulfate concentrations. SMX transformation was normalized by calculating the ratio of SMX concentration at respective sampling day and the initial SMX concentration at day 0, C/C_0_. Controls without cells are included.

**Table S5** Reaction rate constants, intercepts, half-lifes, squared coefficient of determination for SMX transformation by *N. vulgaris*. Tested were 50–650 µM SMX as initial concentrations for a first-order reaction rate.

| **Initial SMX concentration** | **Intercept, *y*** | **Reaction rate constant, *k_SMX_*** | **Squared coefficient of determinant, R^2^** | **Environmental half-life, T_H_** |
| --- | --- | --- | --- | --- |
| 50 µM SMX | 3.782 | 0.1062 | 0.981 | 6.53 |
| 200 µM SMX | 5.138 | 0.0363 | 0.933 | 19.09 |
| 350 µM SMX | 5.733 | 0.024 | 0.925 | 28.88 |
| 500 µM SMX | 6.112 | 0.0258 | 0.974 | 26.87 |
| 650 µM SMX | 6.365 | 0.0217 | 0.924 | 34.48 |

**Fig. S4** SMX transformation activity per cell within the 100 µM SMX and 8–40 mM lactate batch. Displayed is the data for the first 55 days of sampling with available cell densities for normalization.

(a)

(b)

(c)

(d)

(e)

(f)

(g)

(h)

*(Fig. description next page)*

**Fig. S5** Detailed analyses of the cultures shown in Fig. 3 (analysis of the effect of lactate on SMX transformation). Shown are cell numbers (a, b), lactate concentrations (c, d), acetate concentrations (e, f) and sulfide concentrations (g, h). Cultures with 100 µM SMX, 8 mM sulfate and varying concentrations of lactate are shown on left panels (a, c, e, g, red lines). The right panels (b, d, f, h) show the NSC, NCC and sulfide control. Note the different time scale in panels a) and b) to the rest. Cell density was monitored until day 52 when cell aggregate formation occurred which made further cell counting unreliable. The starting cell density was 1.53 x 10^6^ cells mL^-1^. Additional substrates were added at day 149: 16 mM lactate to 8 mM lactate cultures; 0.3 bar H_2_ to 16 mM lactate cultures and 8 mM K_2_SO_4_ to 40 mM lactate cultures.


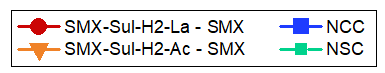

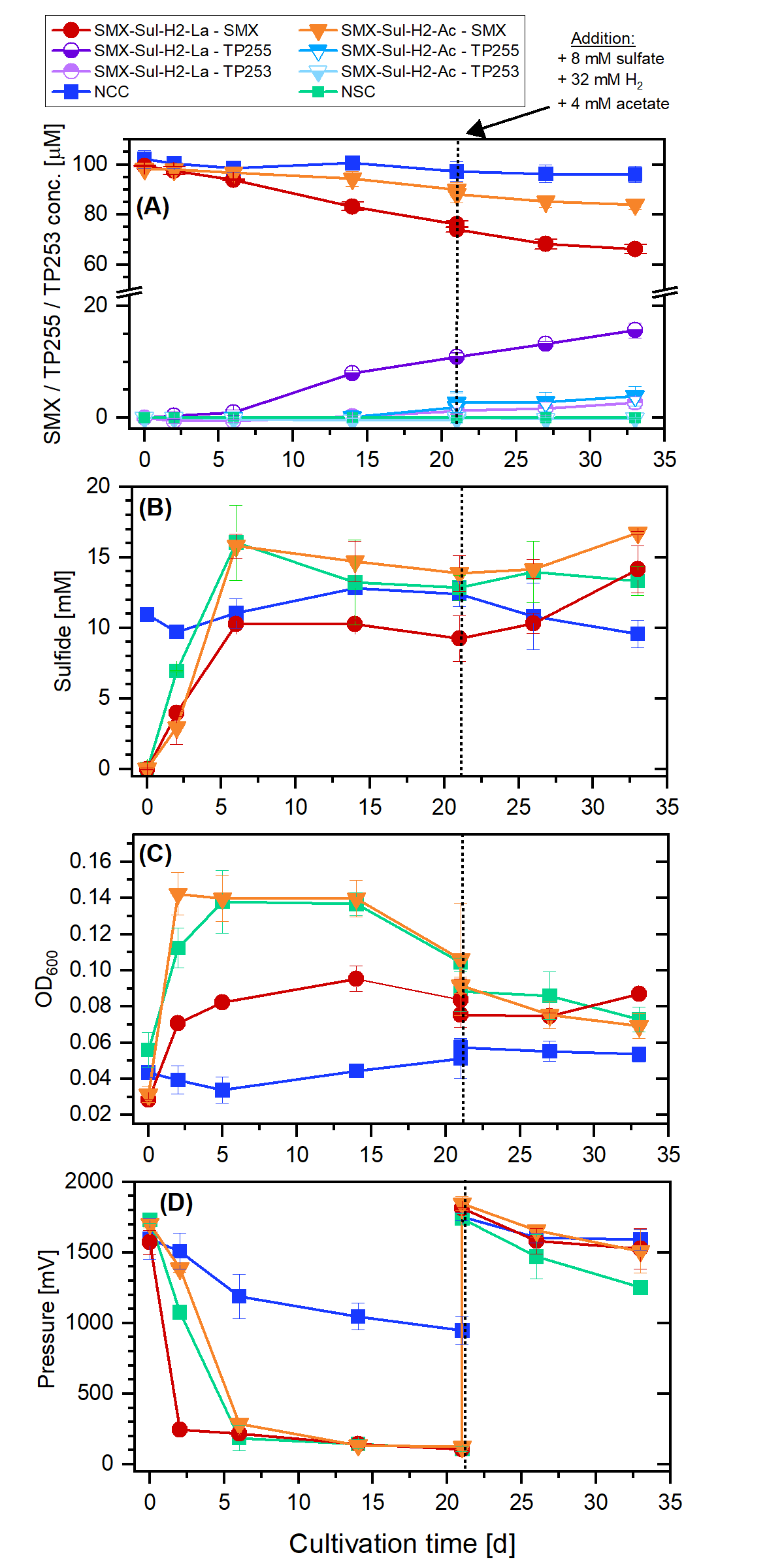


**Fig. S6** Monitored pressure in *N. vulgaris* batch cultures using an analog pressure sensor (MPX5100DP, NXP Semiconductors, Eindhoven, Netherlands) monitoring the headspace pressure within the incubation bottles after over-pressurizing them with pure hydrogen gas. Included is the overhead pressure within the first 21 days of cultivation and after respiking 8 mM sulfate, 32 mM H_2_ (nominal concentration) and 4 mM acetate until day 33 into all cultures. Included are an NCC, NSC and cultures with 24 mM lactate instead of 4 mM acetate*.*


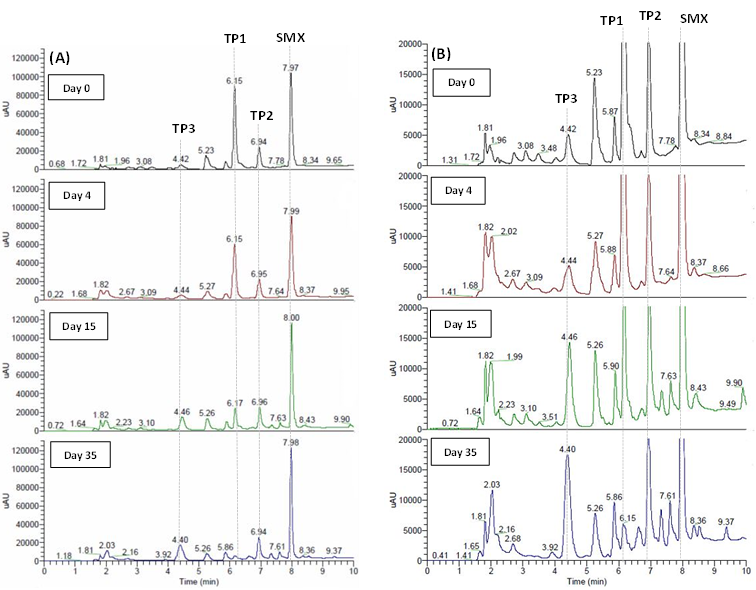


**TP187**

**TP187**

**TP255**

**SMX**

**TP255**

**TP253**

**SMX**

**(a)**

**(b)**

**TP253**

**Fig. S7** Chromatograms obtained by UPLC-DAD at 270 nm to analyze the stability of by *N. vulgaris* formed anaerobic SMX TPs under oxic conditions over time. Shown are (a) different incubation times and (b) a magnification of the same chromatograms to the smaller peaks. Annotated are the peaks for SMX (R_T_ = 7.98 min), TP255 (R_T_ = 6.15 min), TP253 (R_T_ = 6.95 min) and TP187 (R_T_ = 4.44 min).


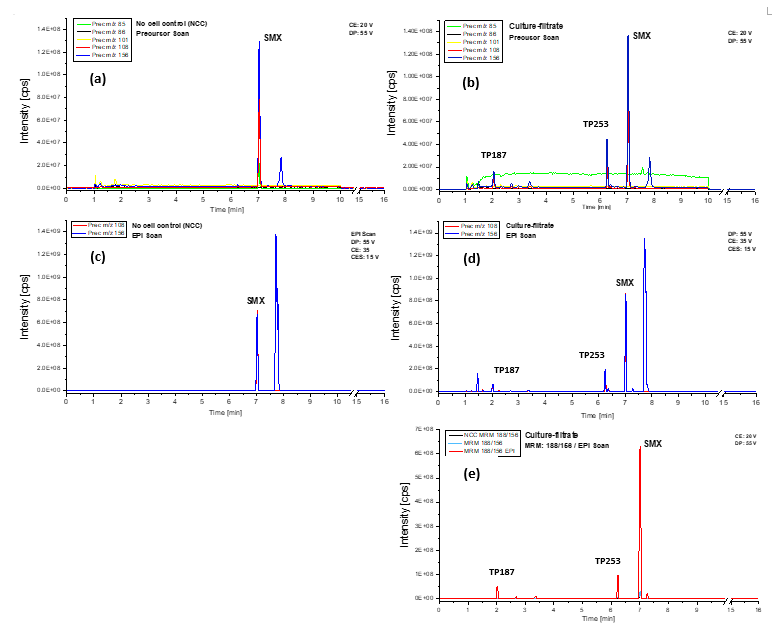


**Fig. S8** Mass spectrometric analysis by LC-MS/MS-QTRAP for specific mass fragments of SMX and its transformation products (TPs). Panels (a) and (c) show results from an NCC in which 50 µM SMX was first anaerobically incubated without cells for several months (Fig. 3, blue lines) and then aerobically incubated for 35 days. Panels (b), (d) and (e) show samples that derive from cultures with 50 µM SMX incubated for several months (Fig. 3, red lines) which were then sampled, sterile filtered and incubated aerobically for 35 days to analyze the stability of TPs. Panels (a) and (b) show precursor ion scans for various known SMX fragments; panels (C) and (D) show Enhanced Product Ion (EPI) scans in which product ions are enriched in an ion trap before analysis for *m/z* values 108 and 156; panel (e) shows an MRM-EPI scan for *m/z* 188/156. Annotated are the signals for SMX (R_T_ = 7.00 min), TP253 (R_T_ = 6.20 min), and TP187 (R_T_ = 2.00 min).


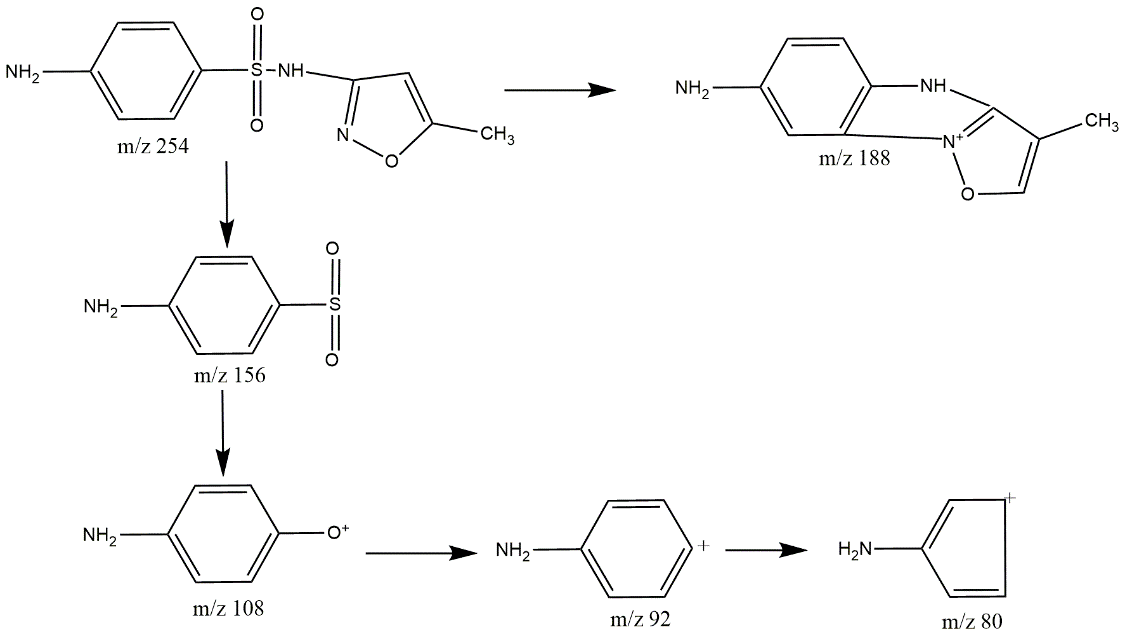

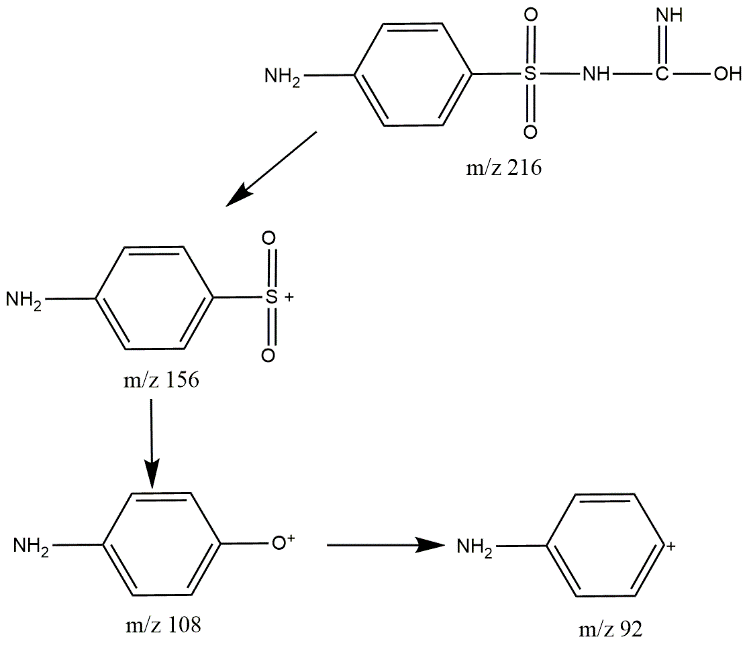

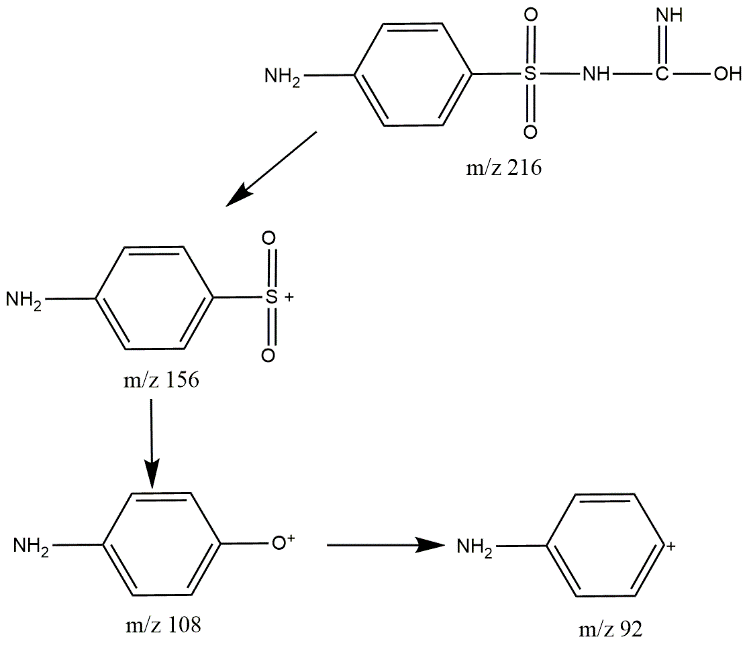

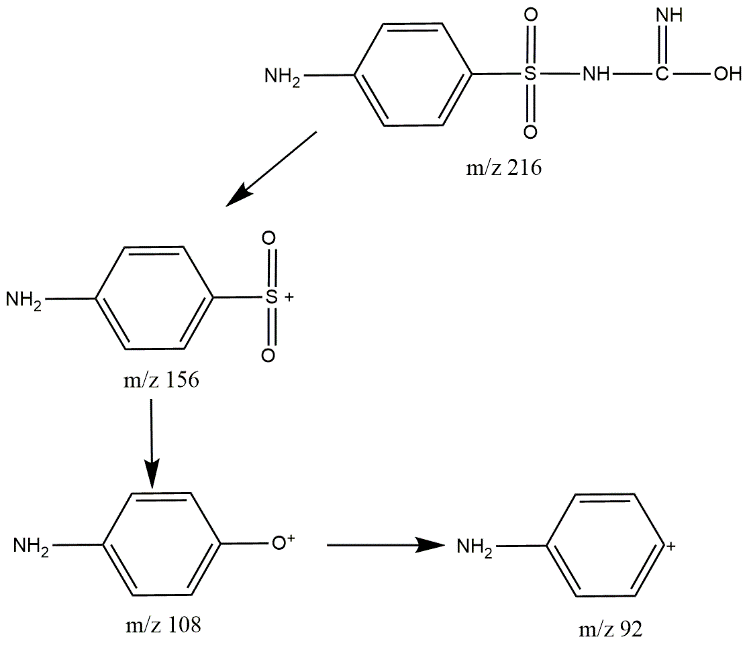


TP188
[M+H]


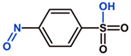


**Fig. S9** Mass spectra obtained by LC-MS/MS-QTRAP analysis of TP187 and its fragments (R_T_: 2.00 min, CE: 35 V, CES: 15 V). Included are hypothesized chemical structures of fragments indicating that TP187 contains an intact aminophenylsulfon substructure.


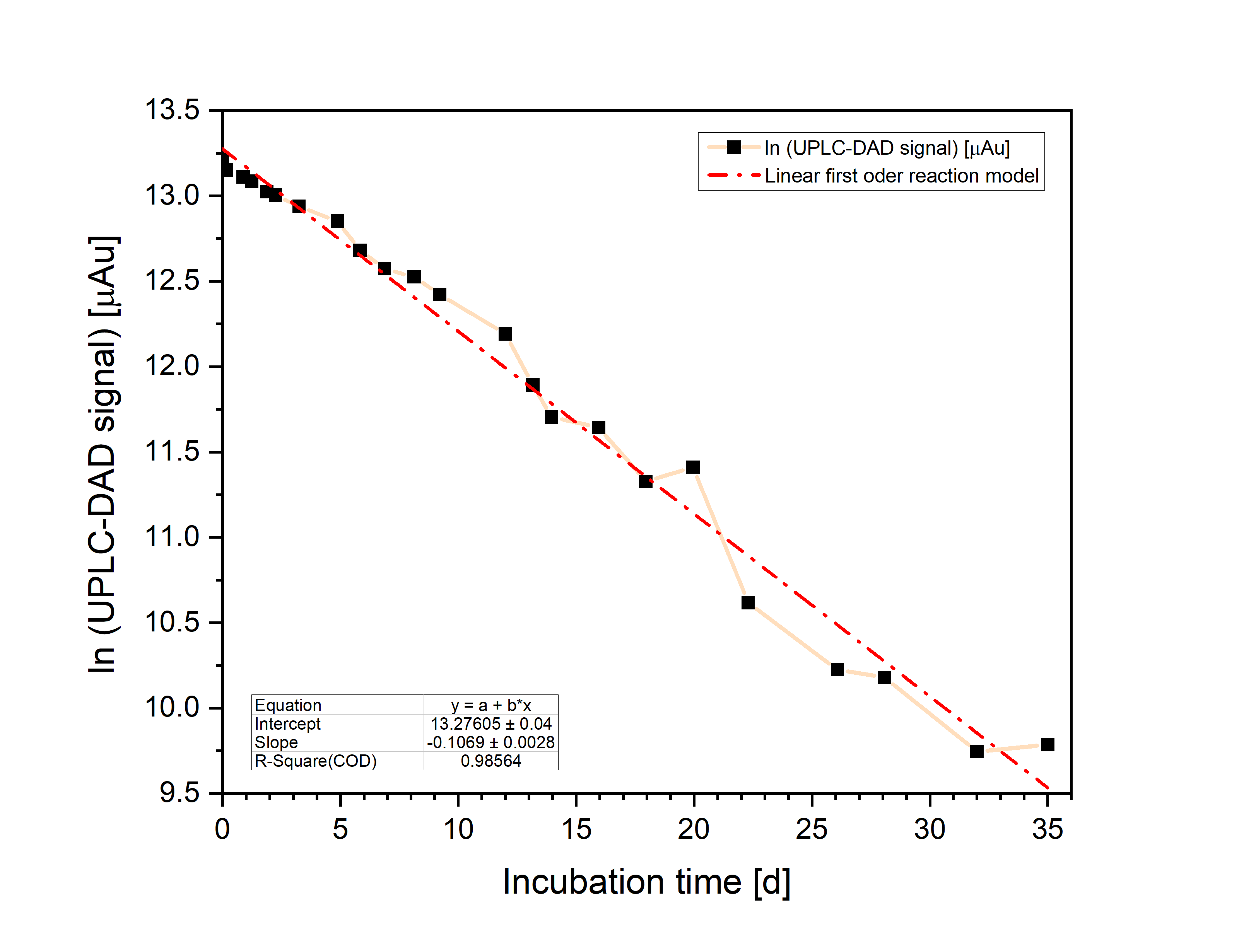


**Fig. S10** Removal of TP255 under oxic conditions based on the measured signal intensity (UPLC-DAD) follows first order reaction kinetics with a reaction constant *k_TP255_* of 0.11 d^‑1^ and a half-life of 6.5 days at room temperature and pH 8.0.

## References

Adrian L, Manz W, Szewzyk U, Görisch H. 1998. Physiological Characterization of a Bacterial Consortium Reductively Dechlorinating 1,2,3- and 1,2,4-Trichlorobenzene. Appl Environ Microbiol 64, 1998: https://doi.org/10.1128/AEM.64.2.496-503.1998

Ding C, Adrian L. Comparative genomics in "Candidatus Kuenenia stuttgartiensis" reveal high genomic plasticity in the overall genome structure, CRISPR loci and surface proteins. BMC Genomics, 2020; 21: 851.

Widdel, F. (1980). Anaerober Abbau von Fettsäuren und Benzoesäure durch neu isolierte Arten Sulfat-reduzierender Bakterien. PhD Thesis, 1980, Georg-August-Universität, Göttingen, Germany.
